# Improving PARP inhibitor efficacy in bladder cancer without genetic BRCAness by combination with PLX51107

**DOI:** 10.1002/1878-0261.70148

**Published:** 2025-11-11

**Authors:** Jutta Schmitz, Anna L. Bartkowiak, Michael Rose, Nora Kolks, Patrick Petzsch, Vandana Solanki, Anne Stoffel, Bianca Faßbender, Leandra Lepping, Julka Volkamer, Karl Köhrer, Marc Seifert, Tokameh Mahmoudi, Tahlita C. M. Zuiverloon, Günter Niegisch, Michèle J. Hoffmann

**Affiliations:** ^1^ Department of Urology, Medical Faculty and University Hospital Duesseldorf Heinrich‐Heine‐University Duesseldorf Germany; ^2^ Center for Integrated Oncology (CIO) Duesseldorf CIO Aachen Bonn Cologne Duesseldorf Germany; ^3^ Institute of Pathology University Hospital, RWTH Aachen University Germany; ^4^ Institute of Pathology University Hospital, University of Ulm Germany; ^5^ German Study Group of Bladder Cancer (DFBK e.V.) Germany; ^6^ Genomics and Transcriptomics Laboratory (GTL), Biological and Medical Research Center (BMFZ) Medical Faculty and University Hospital Duesseldorf, Heinrich‐Heine‐University Duesseldorf Germany; ^7^ Department of Urology, Erasmus MC Cancer Institute Erasmus University Medical Center Rotterdam The Netherlands; ^8^ Department of Haematology, Oncology and Clinical Immunology University Hospital Düsseldorf Germany; ^9^ Department of Pathology Erasmus University Medical Center Rotterdam The Netherlands

**Keywords:** BET inhibitor, biomarker, Bladder cancer, combination therapy, epigenetics, PARP inhibitor

## Abstract

Advanced urothelial carcinoma (UC) requires new therapeutics beyond chemo‐ and immunotherapies. Clinical trials with PARP inhibitors (PARPi), particularly in Cisplatin‐treated UC, yielded limited response. Biomarker‐based patient selection (apart from BRCAness) or combination treatment may increase efficacy. To identify the most suitable PARPi for UC, we compared Olaparib with Talazoparib. RNA sequencing of PARPi‐treated UC lines revealed few common targets and a different impact on immune response. By analysis of experimental and public clinical data, we identified new UC‐specific PARPi response predictors SLFN5, SLFN11, and OAS1. We investigated a new combination treatment using PLX51107, an epigenetic BET protein inhibitor, to increase PARPi efficacy. The Talazoparib + PLX51107 combination had a strong synergistic impact on UC cells and organoids, including Cisplatin‐resistant cells, allowing dose reduction to spare benign cells. Mechanisms of synergism targeted homologous recombination repair, DNA replication, and apoptosis regulation. In conclusion, we suggest Talazoparib treatment of UC to be highly efficacious on all models examined when combined with PLX51107. This new combination treatment allows efficient application of PARPi Talazoparib to all UC patients, independent of Cisplatin pretreatment and genetic BRCAness.

## Graphical abstract

Clinical trials on PARP inhibitors in urothelial carcinoma (UC) showed limited efficacy and a lack of predictive biomarkers. We propose SLFN5, SLFN11, and OAS1 as UC‐specific response predictors. We suggest Talazoparib as the better PARP inhibitor for UC than Olaparib. Its combination with the BET inhibitor PLX51107 overcame resistance, acted synergistically, suggesting a promising, broadly applicable UC treatment strategy. Mechanisms were identified.

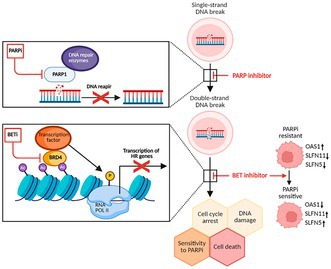

**Abbreviations**
ADC: antibody‐drug‐conjugate
AE: adverse events
BET: bromodomain and extra‐terminal motif protein
BETi: bromodomain and extra‐terminal motif protein inhibitor
BLCA: bladder cancer
CCLE: Cancer Cell Line Encyclopedia
CI: combination index
DAPI: 40,6‐diamidine‐2‐phenylindole
DSB: DNA double‐strand breaks
ER: endoplasmic reticulum
F1CDx: FoundationOne^®^ CDx
Fa: fraction affected
HCF: heart fibroblasts
HDACi: histone deacetylase inhibitor
HRR: homologous recombination repair
ICI: immune‐checkpoint inhibitor
IFN: interferon
ISG: interferon‐stimulated genes
LTT: cisplatin‐resistant UC sublines
NHEJ: non‐homologous end‐joining
Ola: Olaparib
OS: overall survival
PARP: poly (ADP‐ribose) polymerase
PARPi: PARP inhibitor
PBMC: peripheral blood mononuclear cells
PDO: patient‐derived 3D organoids
PI: propidium iodide
PLX: PLX51107
PROTAC: proteolysis‐targeting chimera
qRT‐PCR: quantitative reverse transcription‐polymerase chain reaction
RFS: relapse‐free survival
RT: room temperature
SSB: DNA single‐strand break
Tala: Talazoparib
TBP: TATA box‐binding protein
TCGA: The Cancer Genome Atlas
TMB: tumor mutational burden
UC: urothelial carcinoma
UCC: UC cell lines

## 1 Introduction

Urothelial carcinoma of the bladder (UC) is a frequent cancer type. In 2022, 614 298 new cases were diagnosed worldwide and 220 596 deaths were recorded [1]. Patients with locally advanced muscle‐invasive or metastatic bladder cancer (MIBC) face poor prognosis. Cisplatin‐based chemotherapy has been the standard of care for decades. Recently, new antibody‐based immunotherapies with immune‐checkpoint inhibitors (ICI) and antibody–drug conjugates (ADC) have been approved. However, these treatments are very expensive and not all UC patients are fit enough to receive them. Furthermore, only about 25% of patients respond to ICI therapy and biomarkers predicting response to these treatments are missing [2]. Also, with regard to ADC, recent translational data suggest that a subgroup of patients may not adequately benefit from these treatments with regard to target expression [3]. Thus, improved or new therapeutic approaches are still urgently needed for rising numbers of UC patients and those not eligible for current standard care. Recent data indicate that combination therapies may be more efficient. Moreover, predictive biomarkers would be highly valuable for patient stratification and better‐tailored treatment.

PARP inhibitors (PARPi), in clinical application for breast, ovarian, and prostate cancer, could be one new treatment option for UC, which is characterized by pronounced genomic instability with a high number of genetic alterations and a high tumor mutational burden (TMB). However, in contrast to breast and ovarian cancer, only a small subgroup of UC patients carries genetic alterations in homologous recombination repair (HRR) genes (e.g., *BRCA1/2*, *ATM*, *FANC* genes) [4]. HRR deficiency, also called BRCAness, is known to produce synthetic lethality with inhibitors of poly (ADP‐ribose) polymerase (PARP) that likewise interfere with DNA damage repair [5]. PARP1 is critical for DNA single‐strand break (SSB) repair as it remodels chromatin for recruitment of DNA repair protein complexes. If not repaired, for example, as a consequence of PARP inhibition, SSB convert to DNA double‐strand breaks (DSB). Depending on the cell cycle phase, cells can repair DSB either by template‐guided HRR or error‐prone nonhomologous end‐joining (NHEJ) [6]. In addition to inhibition of PARP enzymatic activity, PARP1 inhibitors can trap the enzyme on DNA, which contributes to antineoplastic effects. PARP trapping potency differs among PARPi, being higher in Talazoparib compared to Niraparib, Olaparib, Rucaparib, or Veliparib [7]. These differences influence the required drug doses, which are lower for Talazoparib [8].

Since BRCAness due to HRR deficiency is common in certain subtypes of breast, ovarian, and prostate cancers, PARPi like Olaparib belong to the standard of care for such entities, applied either alone or in combination [9, 10]. Prior to treatment decision, genetic testing is performed, for example, by the FoundationOne^®^ CDx (F1CDx) assay analyzing genomic alterations in 324 genes including 17 HRR‐associated genes or by OncoPlus [11, 12, 13]. In UC, genetic alterations in HRR genes are uncommon. Some clinical trials have been performed or are ongoing to investigate the use of PARPi for UC [14, 15]. Trials differ in treatment schedule, pretreatment with Cisplatin of included patients, use of either mono‐ or combination therapy (e.g., with ICIs), and inclusion criteria with regard to analysis of genetic alterations in DNA repair genes. While some trials indicated a possible treatment response, especially in rare *BRCA1/2*‐mutated patients, others do not [16, 17, 18]. In unselected patient cohorts, no advantage for progression‐free survival was seen for the treatment regime employing Rucaparib or Olaparib [14, 19]. Also, PARPi mono‐treatment in Cisplatin‐pretreated patients yielded discouraging results [15]. These data highlight the major issues with PARPi treatment approaches for UC: (1) Patients may need to be preselected for efficient personalized medicine, but selection only by HRR gene alterations may not be precise enough and further UC‐specific markers need to be identified and applied. (2) Overall, combination treatments seem to be more efficient. Thus, new approaches for more efficient PARPi‐based combinations are needed and the most suitable PARPi for UC needs to be identified. (3) PARPi resistance mechanisms need to be explored to reveal new strategies how to prevent or reverse PARPi resistance, which may extend the suitability of PARPi to HRR‐competent patients. Beyond genetic alterations, such mechanisms could include altered expression of resistance factors, especially UC‐specific factors. (4) Since UC patients are often pretreated with Cisplatin, new combinations should ideally also be efficient in this setting. All these issues have been addressed by our study.

We aimed at developing a combination treatment for UC based on inhibitors of epigenetic enzymes which ‘episensitize’ toward PARPi by their global effect on chromatin compaction and transcriptional activity. We previously reported that mono‐treatment of UC cell lines (UCC) with PLX51107 (PLX), an inhibitor of Bromodomain and extraterminal motif proteins (BETi) induced a BRCAness phenotype and synergized with PARPi Talazoparib in UMUC3 cells without genetic alterations in HRR genes [20]. These data suggested that epidrug induced BRCAness could extend PARPi efficacy to the majority of UC patients that are non‐HRR‐mutated. As epigenetic readers, BET proteins like BRD4, bridge histone acetylation and transcriptional activity [21, 22]. BRD4, a main target of BETi like PLX, has been found overexpressed in UC tissues and associated with aggressive cancer phenotype and poor survival [23, 24]. BETi are being investigated in clinical trials for other cancers, including the second‐generation BETi PLX that was developed for an improved pharmaceutical profile [25].

In this study, we describe for the first time a synergism of combined treatment with BETi and PARPi in Cisplatin‐pretreated UC models. We compared the well‐characterized PARPi Olaparib (Ola) with second‐generation PARPi Talazoparib (Tala) having higher PARP trapping potency and characterized their cellular and molecular effects in UCC and our in‐house established Cisplatin‐resistant sublines (LTTs). According to our results, we propose Tala as the most suitable PARPi for UC [26]. To characterize the differences in molecular response between both PARPi, which may also underlie the response of UC patients to different PARPi in clinical trials, we performed RNA sequencing and identified the unique and common genes regulated by either PARPi, for example, *SLFN5*. Analysis of own and public data demonstrated that HRR genes included in the F1CDx assay rarely correlated with PARPi response in UCC demanding for other UC‐specific PARP response predictors. Of the discussed PARPi resistance factors [27, 28], we identified *SLFN11* as relevant in UC and newly identified by our own analyses *OAS1* as a PARPi response‐predicting factor in UC. Expression of *SLFN5*, *SLFN11*, and *OAS1* was also significantly correlated with the survival of UC patients in the TCGA cohort.

We found the Tala + PLX combination highly efficient in all investigated UCC, including the Cisplatin‐resistant cell lines and organoids. Thus, we suggest Tala as a suitable PARPi for UC, and our combination treatment would also be applicable to Cisplatin‐pretreated patients in an all‐comer setting, and predictive biomarkers might not be urgently required. Due to the strong synergism, the dosage of the drugs could be reduced to better spare benign cells. Finally, we identified mechanisms underlying the synergism of the drug combination. In conclusion, our study provides important data for the improvement of PARPi‐based therapy of UC and the future design of clinical trials.

## 2 Materials and methods

### 2.1 Cell culture

Urothelial carcinoma cell lines (UCC) were provided by the DSMZ (Braunschweig, Germany) and Dr. B. Grossman (Houston, TX, USA). The corresponding Cisplatin‐resistant sublines (LTT) were in‐house generated as previously described [26]. As control cells, we used commercial HBLAK cells (CELLnTEC, Bern, Switzerland) originating from spontaneously immortalized normal human urothelial cells [29]. THLE‐2 cells were obtained from the ATCC, Heart fibroblasts (HCF) were obtained from Promocell (Heidelberg, Germany; #C‐12375) and cultured according to the manufacturer. Cell lines were regularly authenticated in the past 3 years by DNA fingerprint analysis (STR) and checked for mycoplasma contamination by PCR. All experiments were performed with mycoplasma‐free cells. RRID‐IDs: RT112 (RRID:CVCL_1670), T24 (RRID:CVCL_0554), J82 (RRID:CVCL_0359), HBLAK (RRID:CVCL_JQ59), 253 J (RRID:CVCL_7935), VMCUB1 (RRID:CVCL_1786), UMUC3 (RRID:CVCL_1783), THLE‐2 (RRID:CVCL_3803).

Human bladder tissue for patient‐derived 3D organoid (PDO) cultures was obtained from the Erasmus MC Bladder Cancer Center, Rotterdam, the Netherlands, and the Amphia Ziekenhuis, Breda, the Netherlands. Bladder organoids from biopsies obtained through TURBT or cystectomy were isolated at Erasmus MC Bladder Cancer Center, Rotterdam, the Netherlands, and cultured using methods developed by Mullenders et al. [30] and as further described in [31]. The study was approved by the Institutional Review Board (IRB) of the Erasmus University Medical Center (MEC‐2021‐0354), and all patients provided informed consent.

Peripheral blood mononuclear cells (PBMCs) were isolated from EDTA blood samples using Pancoll gradient (PAN Biotech, Aidenbach, Germany). The study was approved by the Ethics Committee of the University Hospital Duesseldorf (2023‐2667; 2025‐3381) and participants provided informed consent. Cells were cultured and treated for 3 days. All procedures performed were in accordance with the Declaration of Helsinki.

BET and PARP inhibitors were purchased from Hycultec and dissolved in DMSO. 24 h after seeding, cells were treated with drugs or DMSO as the solvent control. After 72 h, the viability of UC cell lines and HBLAK was measured using the MTT assay (Sigma‐Aldrich, St. Louis, MO, USA). For the analysis of organoid viability after treatment, organoids were seeded in 50% BME Cultrex Type 2 (Bio‐techne, Wiesbaden, Germany) and analyzed after 72 h using Cell Titer Glo 3D (Promega, Walldorf, Germany). The viability of further control cells (PBMCs, THLE‐2, HCF) was measured using Cell Titer Glo (Promega). For clonogenicity tests, 1000 cells of the DMSO control were reseeded after 72 h per six wells. The same cell suspension volume was also reseeded for drug‐treated cells. After culturing for at least 12 days, Giemsa staining (Merck Millipore, Darmstadt, Germany) was performed to visualize the long‐term effect of BETi and PARPi and their combinations on cell proliferation.

### 2.2 Calculation of IC_50_ values, drug synergy, and statistics

To determine IC_50_ values, defined concentration ranges of the drugs were tested on the cell lines, and viability was determined either by MTT (Sigma‐Aldrich, Steinheim, Germany) or Cell Titer Glo assay (Promega). IC_50_ values were calculated using GraphPad Prism.

To determine the synergistic effects of combination therapies, compounds were applied as mono‐ and combination treatment at fixed dose ratios based on the IC_50_ values (0.125×, 0.25×, 0.5×, 0.75×, 1×, 1.5×, and 2× IC_50_). DMSO‐treated cells served as solvent controls. The combination index (CI) was calculated using the Chou‐Talalay method and CompuSyn software [32]. The CI value was used to derive the synergy (CI < 1), additive effect (CI = 1) or antagonism (CI > 1) of the combination therapy compared to the single drug treatment [33].

The Combenefit tool was used to analyze synergism on PDO cultures using the Loewe model. The compounds were applied as mono‐ and combination treatments, while DMSO‐treated organoids served as solvent control. Synergy distribution is color‐ and number‐coded (blue and values >0: synergy; red and values <0: antagonism) [34].

For dose–response analyses subsequent to siRNA knockdown, UC cell lines were seeded in 6 wells and transfected the same day using Lipofectamine RNAiMAX (Thermo Fisher Scientific, Dreieich, Germany) and ON‐TARGETplus siRNA SMART pools containing four different siRNAs (Dharmacon, Lafayette, CO, USA; *SLFN11* # L‐027164‐01; *OAS1* #L‐011344‐00; nontargeting #D‐0018110‐10) at a final concentration of 10 nm. On the next day, cells were seeded in 96 wells and treated with inhibitor compounds and incubated for 72 h prior to viability measurements and harvesting RNA for transfection controls.

### 2.3 Flow cytometry

Cell cycle and cell death analyses were performed as already described [35]. Adherent and floating cells were collected 72 h after treatment of the cells with the respective doses. For cell cycle analysis, cells were stained with Nicoletti buffer (50 μg·μL^−1^ propidium iodide (PI), 0.1% sodium citrate, and 0.1% Triton X‐100). Numbers of apoptotic and necrotic cells were determined after incubation of cells with Annexin V‐FITC (Miltenyi Biotec GmbH, Bergisch Gladbach, Germany) in Annexin V binding buffer and PI (2 μg·mL^−1^). Flow cytometric analysis was performed using the Miltenyi MACSQuant^®^ Analyzer and MACSQuantify software (Miltenyi Biotec GmbH).

### 2.4 Protein analysis

Immunoblot analysis was performed with whole cell extracts as described in [36]. Protein extracts were isolated 72 h after treatment. The antibodies used for target detection were PARP1 (Invitrogen #436400; 1:1000), cleaved PARP (Cell Signaling, Danvers, MA, USA #9541; 1:500), SLFN11 (Cell Signaling #34858), OAS1 (Cell Signaling #14498), BIRC5 (Cell Signaling # 2808), BCL2 (Cell Signaling #15071), FANCD2 (Cell Signaling #16323), and α‐Tubulin (Sigma‐Aldrich, Steinheim, Germany #B512; 1:50000). The Rabbit Anti‐Mouse HRP (Dako, Santa Clara, CA, USA #P0260; 1:1000) and the Goat Anti‐Rabbit HRP (Dako #0448; 1:2000) were used as secondary antibodies. Targets were visualized using SuperSignal™ West Femto (Thermo Fisher Scientific, Darmstadt, Germany). Band intensities were quantified using the densitometry tool of the Image Lab software (Bio‐Rad, Feldkirchen, Germany).

Immunocytochemistry was performed as previously described [37]. In brief, cells were seeded on coverslips and treated for 72 h. Cells were fixed with formaldehyde, permeabilized, and incubated in blocking solution. Staining was performed by incubating in primary antibody solution (γH2AX: Cell Signaling #80312, 1:100; RAD51: Millipore #ABE257, 1:500) overnight at 4 °C, followed by a one‐hour incubation with the secondary antibody (Goat Anti‐Mouse IgG (H + L) Alexa Flour 488: Thermo Fisher #A‐11029, 1:250; Goat Anti‐Rabbit IgG (H + L) Alexa Flour 594: Thermo Fisher #A‐11012, 1:500) at room temperature (RT). Nuclei were counterstained with DAPI (40,6‐diamidine‐2‐phenylindole) before mounting (Dako, Santa Clara, CA, USA). Fluorescence microscopy and image analysis were performed at the Advanced Light Microscopy Core Facility (Ad‐Light), Medical Faculty of the Heinrich‐Heine‐University, Düsseldorf. Images were acquired on a confocal laser scanning microscope LSM880, Axio Observer (Zeiss, Oberkochen, Germany) equipped with a Plan‐Apochromat 40×/1.4 NA DIC M27 oil objective (Zeiss), using 8× line averaging at 16‐bit depth, a pixel dwell time of 2.06 μs, and a pixel size of 0.13 μm. Fluorochromes were excited sequentially with 405 nm, 488 nm, and 543 nm lasers, and emission was collected between 410 nm – 513 nm, 493 nm – 630 nm, and 548 nm – 697 nm, respectively.

Image analysis was performed on a HP Z8 Fury GS Workstation (256GB, NVIDIA RTX A5000 GPU) using Zen 2 (blue edition, version 3.11.105.05000) and Intellesis machine learning microscopy software (Zeiss). Prior to segmentation, channels were denoised separately using the Void2Void algorithm (number of epochs: 40, batch size: 64, window size: 5, masking ratio 0.0070). Region classes were segmented individually on denoised images using separate segmentation models allowing for the identification of nuclei, ɣH2AX, and RAD51. Models were trained in single channel mode using 33 basic features without postprocessing. Each image was analyzed for object count and total fluorescence area.

### 2.5 RNA expression analysis

For the analysis of gene expression, UCC were harvested 72 h after treatment. RNA was extracted using the RNeasy mini kit according to the manufacturer's instructions (Qiagen, Hilden, Germany). 1.5 μg of RNA was converted into cDNA using the Maxima H Minus Reverse Transcriptase (Thermo Fisher Scientific, Darmstadt, Hessen, Germany). Quantitative reverse transcription–polymerase chain reaction (qRT‐PCR) was measured with the Luna^®^ Universal qPCR Master Mix (New England Biolabs, Frankfurt, Germany) using a two‐step temperature profile according to the manufacturer's instructions on the LightCycler 96^®^ platform (Roche, Grenzach‐Wyhlen, Germany). Only for *ERCC1*, the SYBR PCR Master Mix (Qiagen, Hilden, North Rhine‐Westphalia, Germany) and a three‐step temperature profile were used. As a reference, the housekeeping gene TATA box binding protein (*TBP*) was included in the analysis. The primer sequences are listed in Table S1.

3′‐RNA sequencing was performed by the BMFZ core facility of the Heinrich‐Heine‐University as described previously [38]. Briefly, total RNA samples were quantified (Qubit RNA HS Assay, Thermo Fisher Scientific, MA, USA) and quality measured by capillary electrophoresis using the Fragment Analyzer and the ‘Total RNA Standard Sensitivity Assay’ (Agilent Technologies, Inc., Santa Clara, CA, USA). Library preparation was performed according to the manufacturer's protocol using the Lexogen^®^ QuantSeq 3′ mRNA‐Seq Library Prep Kit FWD with UMI's. The Input mount was 200 ng total RNA. Bead‐purified libraries were normalized and finally sequenced on the NextSeq 2000 system (Illumina Inc., San Diego, CA, USA) with a read setup of 1 × 100 bp. The BCL Convert Tool (version 3.8.4) was used to convert the bcl files to fastq files as well for adapter trimming and demultiplexing.

CLC Genomics Workbench (version 23.0.5, Qiagen, Venlo, the Netherlands) was used for data analyses of fastq files. Reads of all probes were adapter trimmed and quality trimmed (using the default parameters: bases below Q13 were trimmed from the end of the reads, ambiguous nucleotides maximal 2). For mapping the Homo sapiens (hg38; GRCh38.107) (July 20, 2022), genome sequence was used. After grouping of samples (three biological replicates) according to their respective experimental condition, the statistical differential expression was determined using the CLC Differential Expression for RNA‐Seq tool (version 2.8, Qiagen, Venlo, the Netherlands). Resulting *P*‐values were corrected for multiple testing by FDR and Bonferroni correction. A corrected *P*‐value of ≤0.05 was considered significant. The CLC Gene Set Enrichment Test (version 1.3, Qiagen, Venlo, the Netherlands) was done with default parameters and based on the GO term ‘biological process’ (H. sapiens; May 01, 2021). For differential gene expression the cut‐off was set to 1.5‐fold. Results were submitted to GEO NCBI (GSE285648).

Further analysis and data visualization were performed using Microsoft Excel and GraphPad Prism. Venn diagrams were prepared with the online tool venny 2.0 [39]; available at: https://bioinfogp.cnb.csic.es/tools/venny/index2.0.2.html venny.php. GO group analysis was performed using the online tool DAVID [40].

Public bladder cancer (BLCA) data sets from the Cancer Genome Atlas (TCGA) [41] network including RNASeqV2 data (level 3) of tumor and normal tissue samples were used. RNASeqV2 data from BLCA tumor samples and associated clinicopathological data can be explored and downloaded using the cBio Cancer Genomics Portal (http://cbioportal.org) [42, 43].

### 2.6 Statistical analysis

We used GraphPad Prism to determine significance between the DMSO control and the different treatments using indicated statistical tests with subsequent correction for multiple comparisons. Asterisks denote *P*‐values which were considered significant; *P ≤ 0.05, **P ≤ 0.01, ***P ≤ 0.001.

Survival curves for recurrence‐free survival (RFS) and overall survival (OS) were calculated using the Kaplan–Meier method with log‐rank statistics using IBM SPSS software version 27.0.1.0 (SPSS Inc., Chicago, IL, USA). OS and RFS were measured from surgery until death or relapse (local/distant) and were censored for patients without evidence of death and tumor recurrence at the last follow‐up date.

## 3 Results

### 3.1 Cisplatin‐resistant LTT cells are more sensitive to BETi PLX51107 than parental UCC

**Fig. 1:**
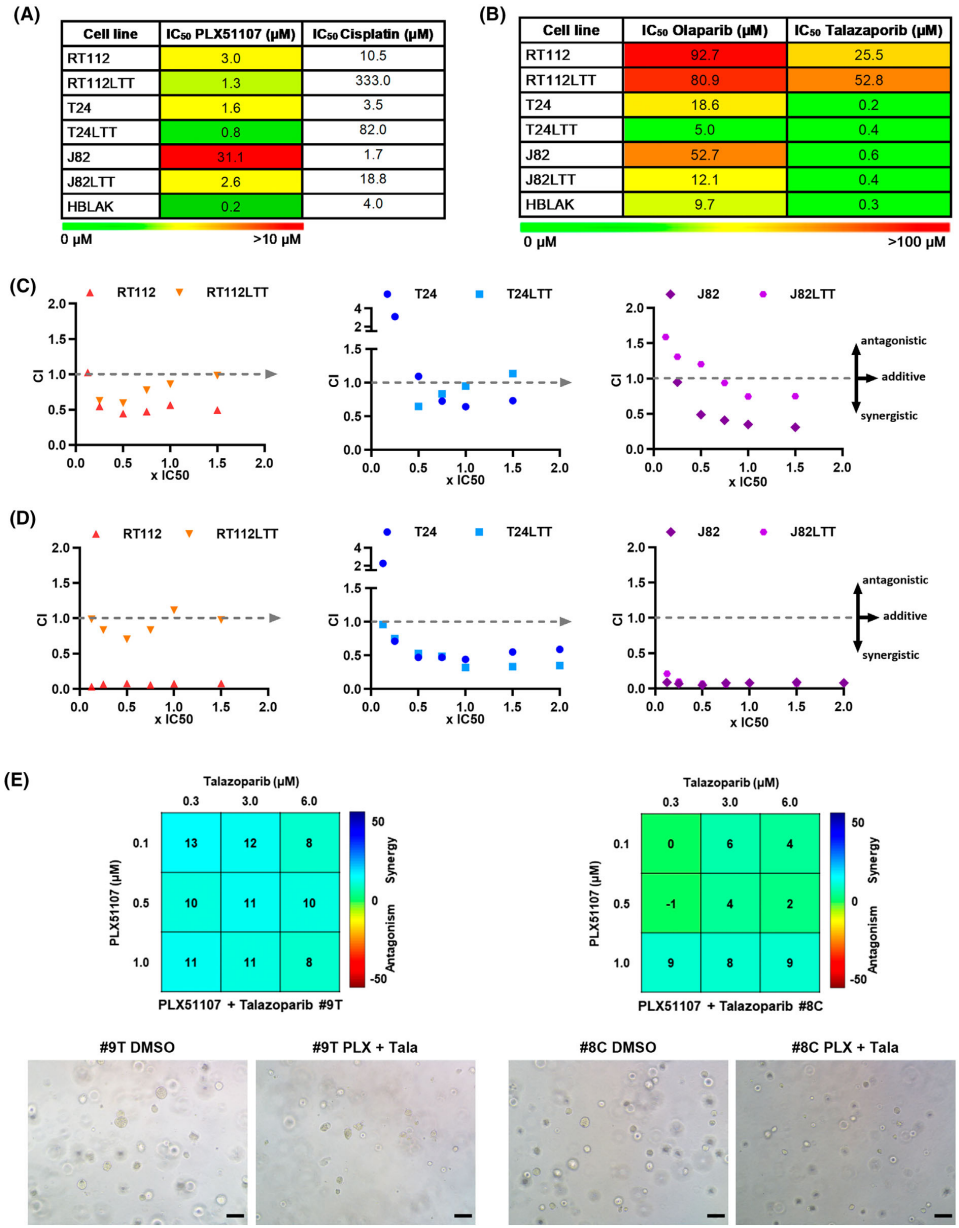
BET inhibitor PLX51107 synergizes with PARP inhibitors Olaparib and Talazoparib allowing dose reduction. (A, B) Color‐coded tables based on half‐lethal IC_50_ dosages for BET inhibitor (BETi) PLX51107 (PLX) (A) as well as PARP inhibitors (PARPi) Olaparib (Ola) and Talazoparib (Tala) (B) show differences in dose–response between parental RT112, T24, J82, and their corresponding Cisplatin‐resistant sublines (LTTs) 72‐h post‐treatment. HBLAK served as a benign uroepithelial control cell line. IC_50_ values for Cisplatin demonstrate Cisplatin resistance of LTT models. (C, D) Chou‐Talalay analysis of the combinations PLX51107 + Olaparib (C) and PLX51107 + Talazoparib (D) with fixed ratios of IC_50_ demonstrated synergism; CI, Combination Index; Fa, fraction affected, CI = 1: additive effect; CI >1: antagonistic effect; CI <1: synergistic effect. (E) Combenefit analysis of synergism for the PLX51107 + Talazoparib combination on bladder cancer patient‐derived 3D organoid cultures. Synergism is indicated by the blue to turquoise color of matrix fields for the indicated dosages. Example microscopy images are displayed. 10× objective, scale bar depicts 100 μm.

Previously, we had characterized the cellular and molecular impact of mono‐treatment with the next‐generation BETi PLX on the two UCC UMUC3 and VM‐CUB1 compared to benign HBLAK cells and found initial evidence for treatment synergism when combined with PARP inhibition in UMUC3 [20]. Since we were seeking to develop a combination therapy with efficacy in the real‐world Cisplatin‐pretreated setting, we performed dose–response analyses for eight further UCC, namely four in house‐generated Cisplatin‐resistant LTT sublines (in order of resistance from top to bottom with difference in IC_50_ values between LTTs and parentals: RT112‐225 μm, 253J‐79.4 μm, T24‐78.5 μm, J82‐17.1 μm, Fig. 1A) and their Cisplatin‐naïve parental UCC (T24, J82, RT112, 253 J) as well as the benign HBLAK cell line in this study. IC_50_ values for the BETi were in the low micromolar range (0.8–3.2  μm), with the exception of J82 and UMUC3 cells that appeared to be rather insensitive (IC_50_ 31.1  μm and 8.8  μm, respectively; Fig. 1A, Fig. S1A). Intriguingly, all Cisplatin‐resistant LTT sublines were significantly more sensitive compared to their parental cell lines (2–12‐fold lower IC_50_ values) suggesting a therapeutic window. HBLAK cells were, however, also sensitive toward the BETi.

### 3.2 Talazoparib is a more potent PARP inhibitor than Olaparib for UCC

Next, we aimed at the identification of the most suitable PARPi for a combination treatment approach with PLX. Initially, we performed dose–response analyses for the well characterized and clinically used PARPi Ola for 10 UCC and benign HBLAK cells. IC_50_ values of UCC for Ola ranged in the intermediate to high micromolar range (5.0–92.7 μm; Fig. 1B, Fig. S1A). RT112 cells appeared resistant toward Ola. Cisplatin‐resistant LTT sublines were again more sensitive tolerating between 12% and 77% lower Ola concentrations compared to their parentals. Unfortunately, the IC_50_ value of benign HBLAK cells was significantly lower compared to most cancer cell lines.

In contrast to Ola, LTTs were no more sensitive than their parental lines to the second‐generation PARPi Tala, but IC_50_ values for all lines were much lower in the nanomolar range (200–600 nM). This finding and the difference between the two PARPi may be significant considering the poor results of clinical trials with PARPi including Cisplatin‐pretreated patients. IC_50_ dosage of benign HBLAK cells was in the same range as that of most UCC (Fig. 1B, Fig. S1A). Thus, Tala was more potent and less toxic. Again, the RT112 cell line pair was the only exception being rather resistant to Tala with IC_50_ values in the high micromolar range (25.5 and 52.8 μm). RT112 cells were thus poorly responding to mono‐treatment with either PARPi.

Since protein‐degrading proteolysis targeting chimera (PROTAC) is considered to be less toxic due to fewer off‐target effects, we also tested the response toward the PARP1 PROTAC SK‐575. However, we found SK‐575 being rather toxic for benign HBLAK cells compared to UCC (IC_50_ dosage: HBLAK 0.6 μm, T24LTT 1.5 μm, J82LTT 2.7 μm), Notably, treatment with the IC_50_ dosage did not result in complete degradation of the PARP1 protein (Fig. S1B, Fig. S8F,G). We thus did not investigate the PROTAC in further treatment combinations.

### 3.3 Combined treatment with PLX and PARPi synergizes in cisplatin‐resistant LTT and UCC

Next, we analyzed cellular responses to combined treatment with the BETi PLX and either Ola or Tala. Fixed ratios of the IC_50_ dosage of PLX and the PARPi were combined and used for treatment of six cell lines, three LTTs, and their parental UCC. Results from viability assays were evaluated for synergistic effects according to the Chou‐Talalay method calculating the affected fraction (Fa) of dead cells vs. the combination index (CI). Combination with Ola revealed that for every analyzed cell line, some dosage points resulted in synergistic effects corresponding to a CI value <1 (Fig. 1C). No large differences were observed between LTT and their parental cells. Instead, the extent of synergism was cell line‐ and particularly dose‐dependent. In J82LTT and T24, synergistic effects were rather limited to certain dosage combinations. Strikingly, both RT112 and RT112LTT cells were the most sensitive to the combination even though they tolerated high Ola dosages in the mono‐treatment underlining that IC_50_ dosage from mono‐treatments does not presage the response to the combined treatment.

The combination of PLX with Tala resulted in stronger and much more robust synergism across all cell lines and independent of dosage (Fig. 1D), suggesting the Tala combination is generally more potent in UC, including the Cisplatin‐resistant setting. These results suggest that the combination could also be efficient in Cisplatin‐pretreated patients that did not clearly benefit from PARPi mono‐treatment in clinical trials. Specifically, J82 cells that were rather insensitive to PLX mono‐treatment responded very strongly to the combination treatment. We had observed similar results earlier for UMUC3 cells that tolerated high PLX dosage in the mono‐treatment but responded strongly to the combined treatment with Tala [20]. Thus, also IC_50_ dosages from PLX mono‐treatments cannot be used as a predictor for response to the combination treatment. Due to the strong synergism of PLX and Tala across all tested cell lines in the clinical application, an all‐comer treatment approach could be reasonable without assessing predictive response markers.

In addition, synergistic effects are advantageous as they should allow to achieve a significant effect on cancer cells with reduced dosages that diminish toxicity to benign cells. Since synergism between PLX and Ola appeared to be dose‐dependent and benign HBLAK cells were sensitive to this combination, we performed extended dose–response analyses of two LTT cell lines (RT112 and T24) for synergistic effects with further nonfixed ratios of IC_50_ dosages. We investigated whether nonequivalent dosages could be more beneficial yielding increased synergism and reduced normal toxicity (Fig. S1C). Indeed, we observed that a reduction to 0.75x IC_50_ Ola combined with 0.25x IC_50_ PLX instead of 1x IC_50_ was highly potent in RT112LTT. Viability of T24LTT was significantly reduced by applying 0.75x IC_50_ Ola in combination with 0.5x IC_50_ PLX.

Likewise, nonfixed ratios were analyzed for the combination of PLX and Tala for two LTT cell lines (Fig. S1D). An even more reduced dosage of 0.5× IC_50_ Tala and 0.25× IC_50_ PLX remained highly potent in both LTT lines and reduced the viability of T24LTT cells more strongly than the Ola combination treatment, again pointing at Tala as the better PARPi for combination treatment.

To demonstrate synergism in a more *in vivo*‐like model, we treated PDO cultures (cisplatin‐resistant (#8C) and cisplatin sensitive (#9T) models) with a matrix of reduced dosage combinations. Again, we could confirm synergism in such models (Fig. 1E). Images of treated cultures indicated apoptosis induction.

When we applied the above‐mentioned reduced dosages for 2D cell lines as mono‐ and combination treatment to benign HBLAK cells, the Tala and PLX combination was better tolerated than the Ola and PLX combination. About 0.5× IC_50_ of T24LTT and T24 was below the Tala IC_50_ dosage of HBLAK. Also, reduced PLX dosage with 0.25× IC_50_ ranged below or near the IC_50_ of benign cells (Fig. S1E). Thus, also with regard to normal toxicity, the combination with Tala was superior to Ola. Accordingly, these reduced dosages (summarized in Table S2) were used to further characterize the cellular and molecular effects of combined BETi/PARPi treatment on two LTTs and their parental lines. Since J82 cells tolerated 10‐ to 20‐fold higher BETi PLX dosages than the other cell lines, we investigated the PLX + Tala combination for the T24 and the J82 reduced dosages. To further investigate normal toxicity, we applied the reduced dosages of these two UCCs mainly containing different PLX amounts (T24: 0.4 μm, J82: 7.8 μm; difference for Tala was only 200 nm) on other benign cell models. To analyze hematological toxicity, we treated PBMCs from different donors (Fig. S2A–C). Dose–response curves revealed that average IC_50_ values of PBMCs were higher than for cancer cells, particularly for Tala (Fig. S2A,B). Concurringly, reduced dosages were tolerated well. Combined treatment with the T24 dosage resulted in 89% vital PBMCs (Fig. S2C).

To investigate further cell types from tissues that may be affected *in vivo*, we investigated heart fibroblasts (HCF; Fig. S2D) and epithelial liver cells (THLE‐2; Fig. S2E). Compared to the reduction of vitality of the according UC cell line (T24 blue and J82 purple bars), HCF cells tolerated the T24 dosage well and even the higher J82 dosage with >75% vitality. Also, liver cells responded much less than the cancer cells. Expectedly, the higher J82 dosage reduced viability more strongly.

### 3.4 Combination treatment with reduced dosages reduced proliferation and induced apoptosis

**Fig. 2:**
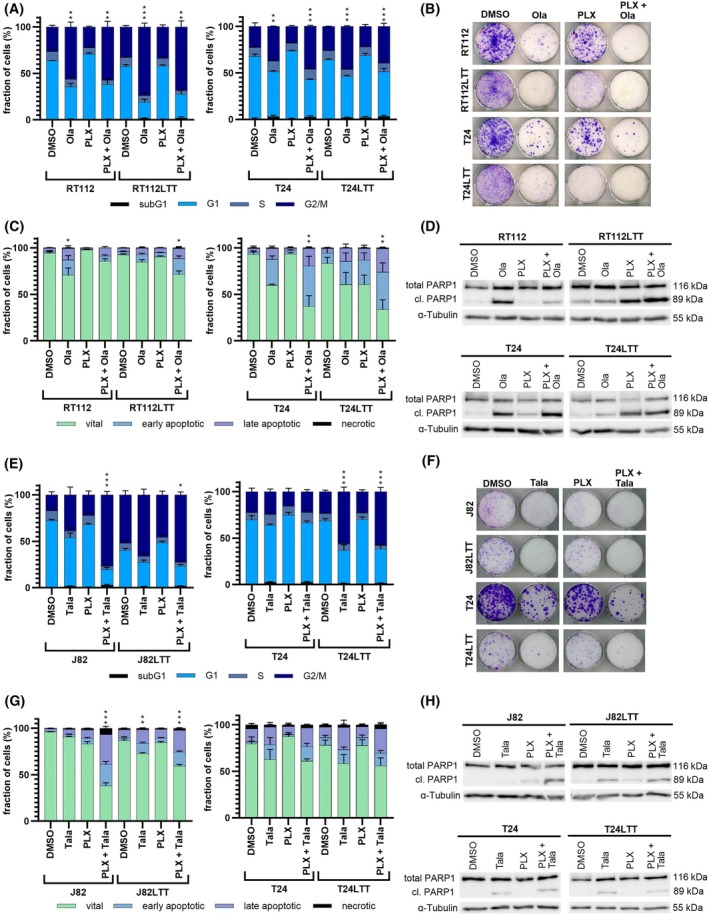
Combination treatment inhibited cell cycle, long‐term proliferation and induced apoptosis. (A) 72‐h post‐treatment with reduced mono or combined dosages of cell line specific IC_50_ of PLX51107 (PLX) and Olaparib (Ola) cell cycle distribution was measured in RT112, T24 and Cisplatin‐resistant sublines (LTT) by flow cytometry compared to DMSO solvent control and averaged over three independent experiments. Significance for G2/M is denoted in comparison to DMSO, ***P ≤ 0.001, **P ≤ 0.01 (mean ± SEM; one‐way ANOVA, Tukey's test). (B) Giemsa staining of the colony formation assay in RT112, T24 and LTTs after treatment with PLX + Ola (*n* = 3). (C) Induction of apoptosis via Annexin V staining and flow cytometry averaged over three independent experiments is displayed. Significance for late apoptotic cells is denoted in comparison to DMSO, **P ≤ 0.01, *P ≤ 0.05 (mean ± SEM; one‐way ANOVA, Tukey's test). (D) Cleaved PARP1 (cl. PARP1) and total PARP1 (D) were determined by western blotting in RT112, T24, and LTTs after combination treatment of PLX + Ola at reduced dosage. α‐Tubulin was detected as a loading control (*n* = 3). (E) 72‐h post‐treatment with reduced mono or combined dosages of cell line specific IC_50_ of PLX and Talazoparib (Tala) cell cycle distribution was measured in J82, T24 and corresponding LTTs by flow cytometry compared to DMSO solvent control and averaged over three independent experiments. Significance for G2/M is denoted in comparison to DMSO, ***P ≤ 0.001, *P ≤ 0.05 (mean ± SEM; one‐way ANOVA, Tukey's test). (F) Giemsa staining of the colony formation assay in J82, T24, and LTTs after treatment with reduced dosages of PLX + Tala (*n* = 3). (G) After 72‐h treatment with reduced PLX + Tala dosages, induction of apoptosis was determined via Annexin V staining and flow cytometry averaged over three independent experiments. Significance for late apoptotic cells is denoted in comparison to DMSO, ***P ≤ 0.001, **P ≤ 0.01 (mean ± SEM; one‐way ANOVA, Tukey's test). (H) Cl. PARP1 and total PARP1 were determined by western blotting in J82, T24 and LTTs. α‐Tubulin was detected as a loading control (*n* = 3).

We applied flow cytometry to analyze the treatment effects on cell cycle and cell death. Cell cycle profiles of combination‐treated cells resembled those of Ola mono‐treated cells, accumulating in G2/M phase (Fig. 2A). Clonogenic assays with lower doses demonstrated reduced toxicity of PLX mono‐treatment, but a strong inhibitory long‐term effect of combined treatment for all four investigated cell lines (Fig. 2B). Annexin V/PI staining demonstrated a strong increase in apoptotic cells elicited by the combination in all cell lines except RT112 parental cells (Fig. 2C). Concurringly, PARP1 cleavage as an indicator of apoptosis could be detected by western blot analysis, particularly in LTT sublines (Fig. 2D, Fig. S8A,B).

Reduced PLX + Tala dosages were likewise applied. Profiles of combination‐treated cells resembled again those of PARPi mono‐treated cells. PLX unresponsive J82 cells responded exceptionally strong to the combined treatment and displayed significant increased numbers in G/2 M compared to the mono‐treatments (Fig. 2E). Clonogenic assays demonstrated significant growth‐inhibitory impact on all four investigated cell lines (Fig. 2F). Apoptosis induction was significantly increased by combined treatment compared to mono‐treated cells in all four cell lines (Fig. 2G). Accordingly, PARP1 cleavage was detected (Fig. 2H, Fig. S8C,D).

### 3.5 Identification of UC‐specific PARPi response marker

Since we had observed that some UCC responded rather poorly to Ola and that RT112 cells were resistant toward both PARPi, we were seeking to identify UC‐specific PARPi response factors. One set of markers that is commonly used for decision taking, for example, for treatment of prostate cancer patients with PARPi is included in the Foundation One^®^ CDx genetic analysis companion diagnostic assay. Thus, we compared public data for UC and breast cancer on mutational background and expression of the 17 HRR‐associated genes included in this assay (*BRCA1*, *BRCA2*, *BRIP1*, *FANCA*, *FANCC*, *FANG*, *FANCL*, *MRE11*, *NBN*, *PALB2*, *POLD1*, *RAD51*, *RAD51B*, *RAD51C*, *RAD51D*, *RAD54L*, *XRCC2*; Figs S3, S4) and Ola response using cBioPortal. Breast cancer cell lines commonly harbored genomic alterations like mutations and particularly deep deletions or amplifications. Apart from the *BRCA* genes (*BRCA1* alterations in 22% of cases, *BRCA2* 29%) seven other genes carried alterations in ≥10% of cases (Fig. S3). Concurringly, many cell lines had low IC_50_ values for Olaparib. In contrast, in TCGA UC tissues, alterations of HRR genes were infrequent; *BRCA2* was the only gene altered in more than 10% of cases (12%, BRCA1 6%; Fig. S4A). Thus, PARPi mono‐treatment would be expected to be only efficacious in a small group of UC patients harboring genetic HRR alterations, in accordance with observations in clinical trials [14, 15]. HRR genes were also less frequently altered in UC cell lines; *BRCA1*, *MRE11*, and *RAD51B* were most frequently affected (11%, 13%, 11%; Fig. S4B). Concurringly, a number of UCC had high IC_50_ values for Ola, also genetically wild‐type RT112 cells, which were also resistant in our dose–response analyses. However, cell lines with genetic alterations in individual HRR genes, also the *BRCA* genes, had not necessarily low IC_50_ values for Ola. Thus, public data confirmed our results on PARPi unresponsiveness of RT112 cells and that PARPi mono‐treatment may only be suitable for a small subgroup of UC requiring biomarker stratification that is not only based on HRR genes. Further, our results on dose response of 10 UCC toward Ola and Tala demonstrate that differences in PARPi response not only rely on genetic background. While T24 and J82 had intermediate IC_50_ for Ola, these were similarly sensitive to Tala as other UCC. To consider not only genetic alterations but also gene expression—which is the analyte used for molecular subtyping—we correlated our own expression data of six HRR genes (*BRCA1*, *BRCA2*, *FANCD2*, *RAD51*, *RAD51B*, *RAD51C*) with dose response of our cell lines (Fig. S5). Positive correlations between low expression and low IC_50_ values would be expected. However, we did not find significant positive correlations for Ola with the investigated HRR genes (Fig. S5A). Instead, Tala response correlated strongly positive with expression of *BRCA1* (Pearson *r* = 0.80, *P* = 0.01) and *FANCD2* (Pearson *r* = 0.90, *P* = 0.01; Fig. S5B). Obviously, further UC‐specific PARPi response factors in addition to those in the Foundation One^®^ CDx HRR panel are needed as predictive biomarkers for response to PARPi mono‐treatment.

**Fig. 3:**
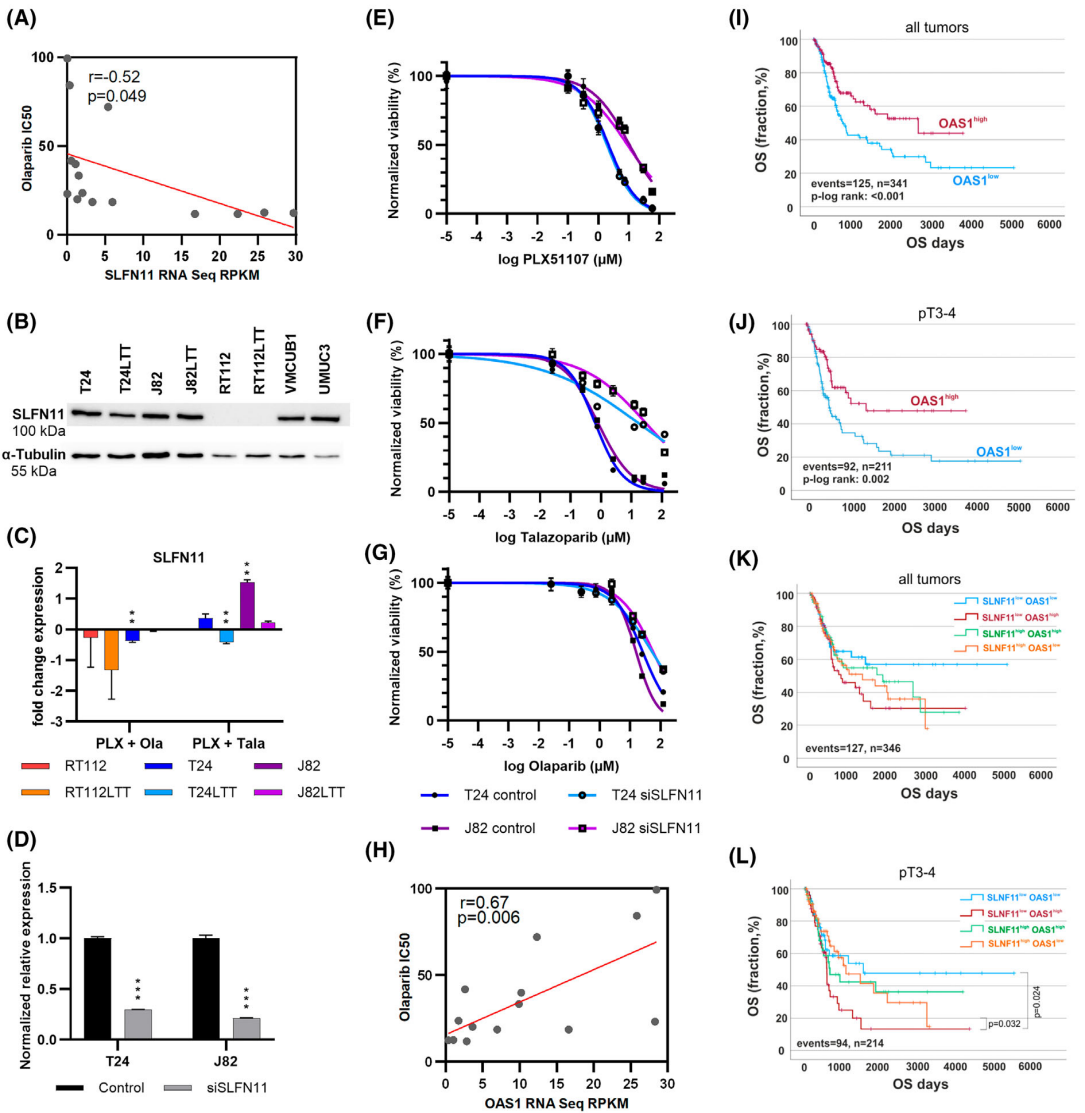
Analyses of new urothelial carcinoma‐specific biomarkers for PARP inhibitor response. (A) Public results (cBioPortal) from dose–response analyses of urothelial carcinoma cell lines (UCCs) for Olaparib (Ola) were correlated with gene expression data for *SLFN11*. Pearson coefficient (R) and *P*‐value are denoted. (B) SLFN11 protein level was determined in indicated UCC by western blotting. α‐Tubulin was detected as a loading control (*n* = 3). (C) Expression changes of *SLFN11* after treatment were detected by qRT‐PCR. *TBP* was measured as a housekeeping gene and used for normalization. Fold change expression (log_2_) was calculated vs DMSO control (set to 0). Significance is denoted in comparison to DMSO **P ≤ 0.01 (mean ± SEM; *n* = 3; two‐way ANOVA, Tukey's test). (D) siRNA knockdown efficacy was confirmed by qRT‐PCR for *SLFN11* compared to non‐targeting control, ***P ≤ 0.001 (mean ± SEM; *n* = 3; two‐way ANOVA, Tukey's test). (E–G) Subsequent dose response was determined for PLX51107 (E), Talazoparib (F), and Olaparib (G). Normalized viability to DMSO control was plotted against logarithmic concentration (mean ± SEM; *n* = 3). (H) Public results (cbioportal) from dose–response analyses of UCCs for Ola were correlated with gene expression data for *OAS1*. Pearson coefficient (r) and *P*‐value are denoted. (I, J) Kaplan–Meier survival curves show overall survival (OS) of bladder cancer patients from the TCGA BLCA cohort with high *OAS1* mRNA expression (>median expression; red curve) compared to low *OAS1* mRNA expression (≤median expression; blue curve) for all tumors (I) as well as classified by pT (J) based on TCGA BLCA data sets. (K, L) Kaplan–Meier plots illustrate OS in relation to combinatorial expression patterns of *OAS1* and *SLFN11* for all tumors (K) as well as classified by pT (L) using TCGA BLCA data sets.

For other cancer types, putative PARPi resistance factors have been discussed in the literature. Genetic or expression loss of *SLFN11*, *WRN*, *SMARCAL1*, *EZH2* or of a functional shieldin complex (*SHLD1/2/3*, *REV7/MAD2L2*, *RIF1*, *TP53BP1*) may underlie PARPi resistance. Loss of *PTEN*, *MRE11*, or specific members of the ABC drug transporter family may contribute to PARPi sensitivity [28]. Our analysis of public data revealed that all mentioned genes are rarely affected by genetic alterations in TCGA UC tissues (Fig. S6A). UCC that were rather resistant to Ola (Scaber, RT4, RT112) harbored alterations in *MRE11*, *ABCA1*, *ABCG2*, *MAD2L2*, and *WRN* (Fig. S6B). *MRE11* expression was increased in Ola‐resistant UCC, while the expression of several other genes was rather reduced (Fig. S6C). Correlating the expression of the genes mentioned above with Ola response using cBioPortal, we found a significant negative association only for *SLFN11*, suggesting that low *SLFN11* expression corresponds to high IC_50_ values for Ola (Pearson *r* = −0.52, *P* = 0.0049; Fig. 3A). Concurringly, our western blot analysis revealed that PARPi‐resistant RT112 did not display SLFN11 protein (Fig. 3B, Fig. S8E). Intriguingly, SLFN11 was induced by the Tala combination treatment, but not by the Ola combination (Fig. 3C).

To further validate *SLFN11* as a prediction marker, we performed siRNA knockdown of *SLFN11* (Fig. 3D) and subsequently measured dose response to mono‐treatments (Fig. 3E–G). As a control and as expected, the response to PLX remained unchanged. Concurring with the above correlation data, we found that *SLFN11* knockdown rendered T24 and J82 more resistant toward both PARPi and particularly strong to Tala treatment (Fig. 3F). IC_50_ values for Ola shifted 2–3.6‐fold (T24 and J82, respectively), while Tala values shifted 26.1–37.2‐fold (T24 and J82, respectively). Thus, *SLFN11* abundance may predict the response to PARPi mono‐treatment in UC. Further validation in clinical trials with sufficiently high patient numbers is needed.

Next, we used our own data on gene expression differences between PARPi‐resistant RT112 and other PARPi‐responsive UCC to identify further candidates for PARPi response biomarkers in UC. These were then correlated with Ola response of UCC using cBioPortal. We found significant positive correlations between good Ola response and expression of *OAS1* (Pearson r = 0.67, *P* = 6.17e‐3) (Fig. 3H). Thus, increased *OAS1* levels may correlate with PARPi resistance. Concurringly, less responsive UCC—particularly RT112, displayed high OAS1 protein levels in western blots, while other UCC were negative (Figs S7A, S8H). Further, *OAS1* was very strongly downregulated by both PARPi combinations in all cell lines contributing to synergism (Fig. S7B). siRNA knockdown of *OAS1* slightly sensitized cells to PARPi; Tala IC_50_ of T24 *OAS1* knockdown cells was 200 nm lower compared to control cells (Fig. S7C,D).

Thus, we propose *OAS1* as a further UC‐specific predictor for PARPi response that also seems to underlie treatment synergism. By analyzing publicly available TCGA data of UC patients, we also found *OAS1* expression significantly correlated with patients' outcomes (Fig. 3I,J). Overall, high *OAS1* expression correlated significantly with longer OS across all tumor samples (*P* < 0.001, median OS: 2641 ± 899 days, 95%‐CI 879 to 4403 days) compared to those with low expression (median OS: 739 ± 92 days, 95%‐CI 560 to 918 days). Stratifying this data set by tumor stage revealed that high *OAS1* expression was significantly associated with better OS in stage pT3/4 tumors (*P* < 0.01). Interestingly, nonparametric Spearman rank correlation demonstrated a slight but significantly inverse correlation between *OAS1* and *SLFN11* mRNA expression (*r*: −0.109, *P* = 0.027) in the TCGA BLCA data set suggesting a putative mechanistic link between both affecting PARPi response (Fig. 3K,L). Among patients with low *SLFN11* expression, survival was significantly strongly dependent on *OAS1* expression levels (*P* = 0.024), with low *OAS1* expression correlating with the best prognosis of all 4 groups. Patients with advanced UC with high *SLFN11* and low *OAS1* expression would face poorer prognosis (Fig. 3L, orange curve), but might be sensitive to PARPi mono‐treatment according to our data. Thus, we propose to analyze the predictive power of *OAS1* potentially in close association with *SLFN11* in clinical trials for PARPi treatment of UC.

### 3.6 Olaparib and Talazoparib have unique downstream targets in UC

**Fig. 4:**
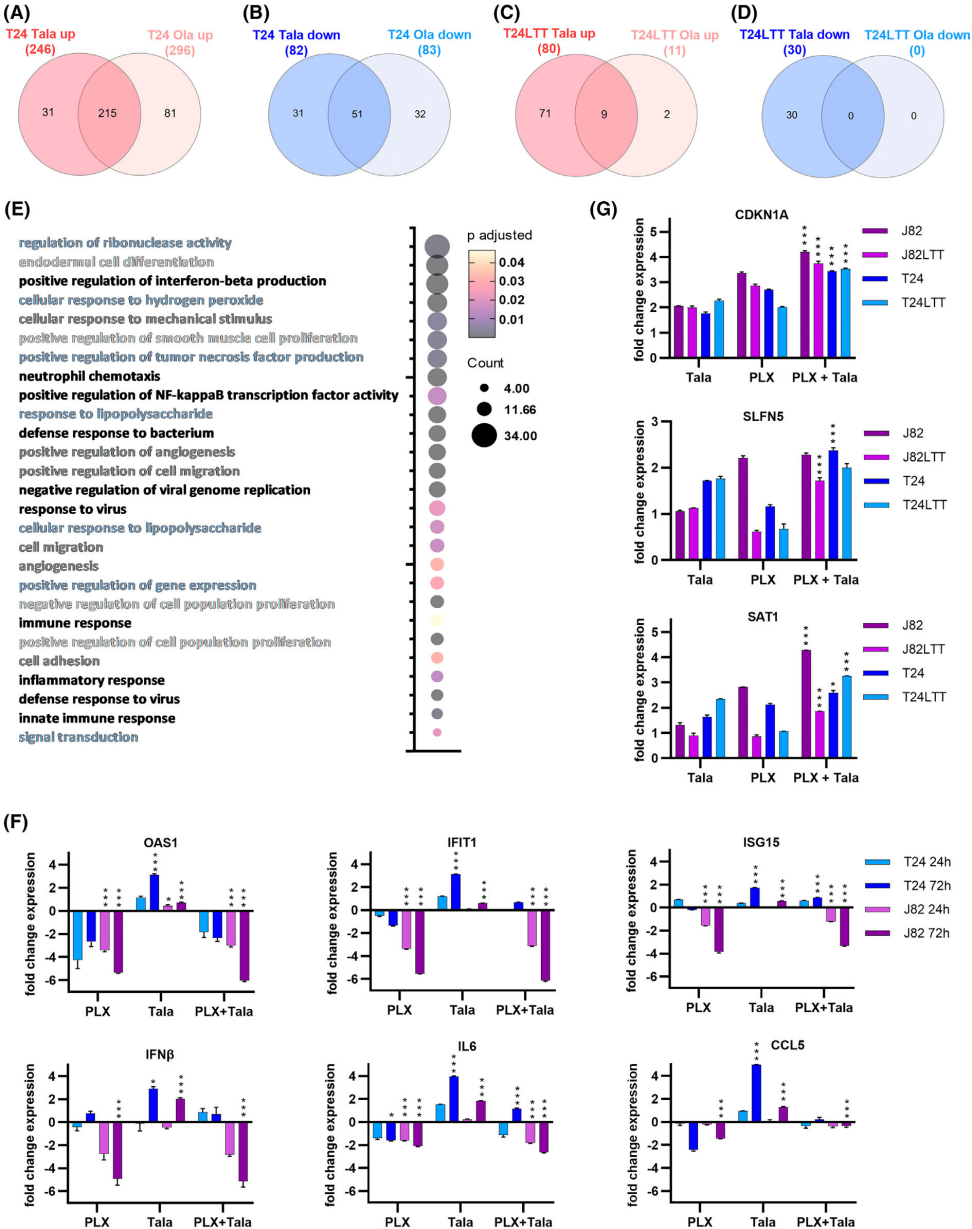
Molecular response of urothelial carcinoma cell lines toward PARP inhibitors was characterized by RNA sequencing. (A–D) Significantly differentially expressed genes (FC ≥ 1.5, Bonferroni adjusted *P* ≤ 0.05) for either indicated condition were compared using Venn diagrams (Venny 2.0) to identify commonly and uniquely up‐ (red) and downregulated (blue) genes between both PARP inhibitors (PARPi) in the T24 cell line pair. (E) 215 commonly upregulated genes by both PARPi in T24 cells were subjected to gene ontology (GO) analysis (DAVID tool) to assign cellular processes that were significantly affected. Bubble plot gives the number of genes enriched for the indicated processes (count); color code denotes false discovery rate (FDR) adjusted *P*‐value. Interrelated processes are marked by the same color for process names. (F) Results of qRT‐PCR validation for cGAS‐STING signaling components 24 h and 72 h after indicated treatment. *TBP* was measured as a housekeeping gene and used for normalization. Fold change expression (log_2_) was calculated vs DMSO control (set to 0). Significance is denoted in comparison to DMSO, ***P ≤ 0.001, *P ≤ 0.05 (mean ± SEM; *n* = 3; two‐way ANOVA, Tukey's test). (G) Results of qRT‐PCR validation for three genes commonly induced in all UCCs by indicated treatment condition. *TBP* was measured as a housekeeping gene and used for normalization. Fold change expression (log_2_) was calculated vs DMSO control (set to 0). Significance is denoted in comparison to mono‐treatments, ***P ≤ 0.001, *P ≤ 0.05 (mean ± SEM; *n* = 3; two‐way ANOVA, Tukey's test).

We further sought to identify genes that were UC‐specifically regulated by PARPi and to elucidate differences in molecular response to either PARPi that might also underlie the response of patients toward the different mono‐treatments. To this end, we performed RNA sequencing of Ola‐ and Tala‐treated T24 and T24LTT cells. We found comparable numbers of up‐ and downregulated genes in T24 cells treated with Tala or Ola (Fig. 4A,B), but its Cisplatin‐resistant LTT subline responded with a lower number of differentially expressed genes after either treatment. The lowest number of genes was deregulated by Ola in T24LTT (Fig. 4C,D).

Low numbers of genes were commonly affected by the two PARPi. About 215 genes were commonly induced by both PARPi in T24, while 51 were commonly downregulated (Fig. 4A,B; Tables S3, S4). Overlap between PARPi in T24LTT was much smaller (Fig. 4C,D). However, seven genes were commonly induced by both PARPi in both cell lines, namely, *SLFN5*, *SAT1*, *FST*, *IFIT2*, *IL1B*, *INHBA*, and *TNFAIP3*. GO enrichment analysis of commonly PARPi‐induced genes in T24 (*n* = 215) revealed enrichment mostly for processes related to immune response, cytokines, cGAS‐STING signaling, and cell stress. Further processes involved regulation of migration, signal transduction, and proliferation (Fig. 4E). Since cGAS‐STING activation is currently discussed to be advantageous for improved response to ICI treatment, we performed qRT‐PCR validation for cGAS‐STING signaling components (Fig. 4F). Indeed, we could prove that Tala mono‐treatment induced expression of all six investigated components, particularly strong in T24 cells (*OAS1*, *IFIT1*, *ISHG15*, *IFNβ*, *IL6*, and *CCL5*). Since basal expression levels were higher in J82 cells compared to T24 (Fig. S7E), these cells could not further increase expression that much. Interestingly, expression of cGAS‐STING components was reduced by the PLX+Tala combination treatment.

Since Tala appeared to be the better combination partner for PLX, we investigated further UCC (J82, J82LTT, RT112) after Tala treatment by RNA sequencing. *SLFN5*, *SAT1*, and *CDKN1A* were induced by Tala in all five investigated cell lines (T24, T24LTT, J82, J82LTT, RT112) and thus seem to be UC‐specific Tala response marker (Table S5). Intriguingly, PARPi‐resistant RT112 cells displayed the most genes regulated by Tala exclusively in this cell line (Table S5). Induced genes were enriched for apoptosis regulators and downregulated genes for DNA replication, cell division, and cell cycle checkpoints.

The qPCR validation of the commonly PARPi induced *CDKN1A*, *SLFN5*, and *SAT1* genes confirmed that all were strongly induced by Tala treatment and even further increased by the combination treatment in LTTs and parental cells (Fig. 4G). Thus, these common PARPi regulated genes also underlie the synergism of the Tala combination and contribute to increased DNA damage, cell cycle arrest, and death. Analysis of public TCGA data demonstrated that *SLFN5* predicted OS (*P* = 0.005) and RFS (*P* = 0.034) in pT1/2 bladder tumors (Fig. S6D), that is, increased *SLFN5* expression was associated with an unfavorable prognosis (mean OS: 1727 days and mean RFS: 1858 days) compared to bladder cancers characterized by low *SLFN5* expression (mean OS: 2756 days and mean RFS: 2978 days).

Comparison of genes regulated in T24 cells by Ola or Tala also revealed some genes that were uniquely altered by either PARPi (Table S4). Interestingly, uniquely Ola‐induced genes were associated with immune response, particularly antigen presentation, which may contribute to treatment benefit when combining Ola with ICI. CD274 encoding PD‐L1 was induced by Tala in T24 but not by Ola and not in J82 and RT112 cells.

### 3.7 Mechanisms underlying synergism of combination treatment

**Fig. 5:**
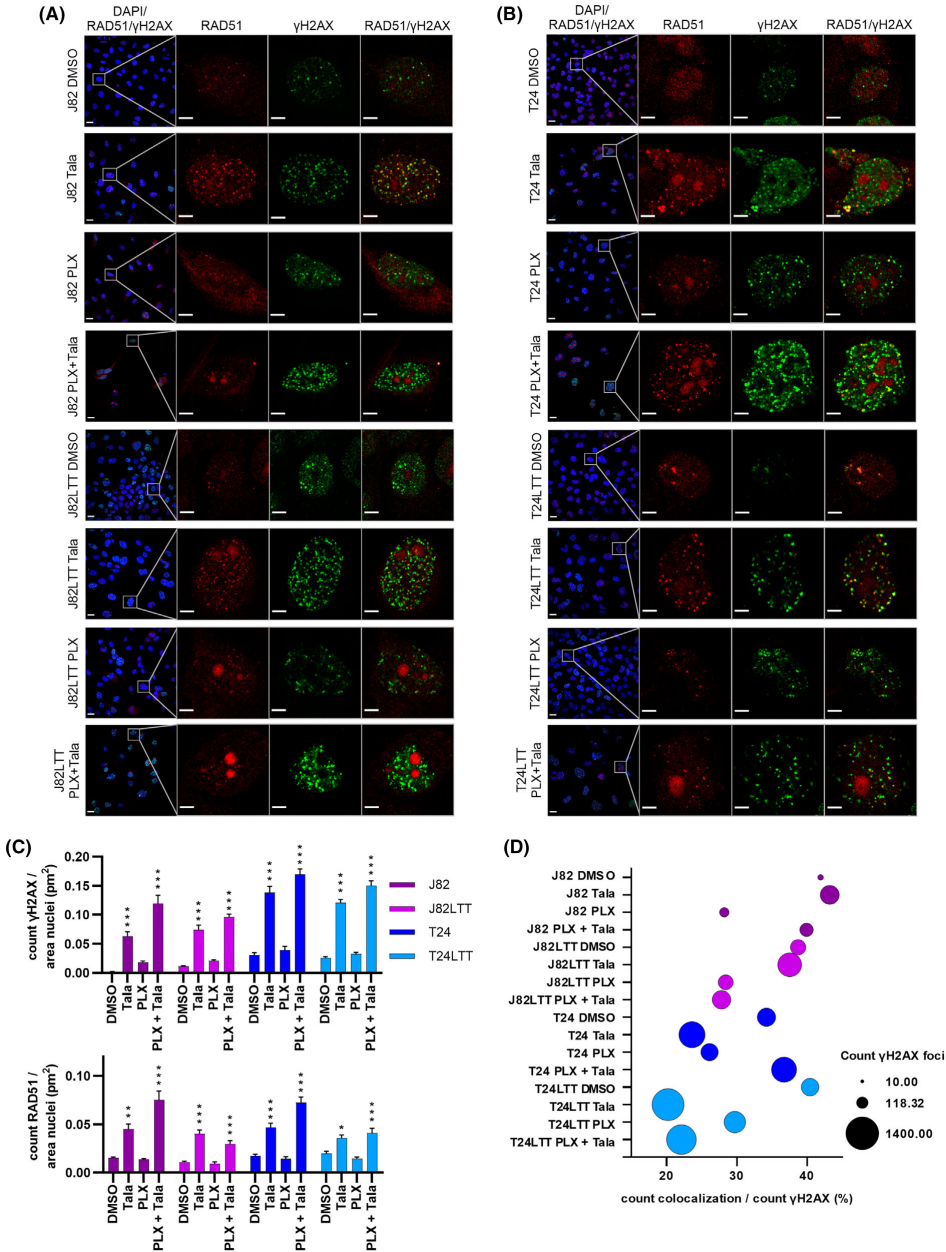
Combination treatment induced DNA double strand breaks remained unrepaired. (A, B) ɣH2AX (green) and RAD51 (red) were antibody stained for detection of DNA double‐strand breaks (DSB). DAPI (blue) was used to counterstain nuclei. DMSO served as the solvent control. Channel overlay of the three channels is shown in the merge column on the left of each panel, while colocalization of ɣH2AX and RAD51 is shown on the right. The indicated treatment with reduced dosages of PLX51107 (PLX), Talazoparib (Tala), a combination of PLX and Tala, and DMSO as the solvent control was applied for 72 h to J82 and J82LTT (A) and T24 and T24LTT (B). The white square in the merged picture shows the zoomed‐in cell. 40× objective, scale bar depicts 20 μm in the overview image and 5 μm in the cropped images of representative cells. (C) AI‐supported quantification of ɣH2AX and RAD51 foci per nucleus area is displayed. Significance is denoted in comparison to DMSO, ***P ≤ 0.001, **P ≤ 0.01, *P ≤ 0.05 (mean ± SEM; *n* = 8; one‐way ANOVA, Tukey's test). (D) Artificial intelligence‐supported quantification of ɣH2AX and RAD51 colocalization is displayed as a bubble plot. The size of bubbles denotes the amount of ɣH2AX foci; the localization of the bubble on the x‐axis denotes the extent of their colocalization with RAD51 foci needed for repair. Different cell lines are color coded as usual (mean values; *n* = 8).

Having observed earlier that PLX induced DSB [20], we investigated whether mono‐treatment also induced DSB in Cisplatin‐pretreated cell lines and the effect of the combination treatment by immunocytochemistry staining and subsequent AI‐based quantification (Fig. 5A,B). PLX and even more Tala mono‐treatment induced ɣH2AX foci both in LTTs and parental cells. Combination treatment resulted in increased numbers of DSB foci, particularly in T24 and T24LTT. Also, the numbers of RAD51 foci were increased by Tala mono‐treatment and the combination (Fig. 5C). Interestingly, the amount of colocalized ɣH2AX and RAD51 foci was reduced by combination treatment in J82 and J82LTT suggesting that cells were not able to recruit RAD51 extensively enough to sites of DSB for repair which may contribute to the particular synergism observed in the J82 cell line pair (Fig. 5D). Instead, RAD51 rather accumulated in a few large foci in the nucleoli.

**Fig. 6:**
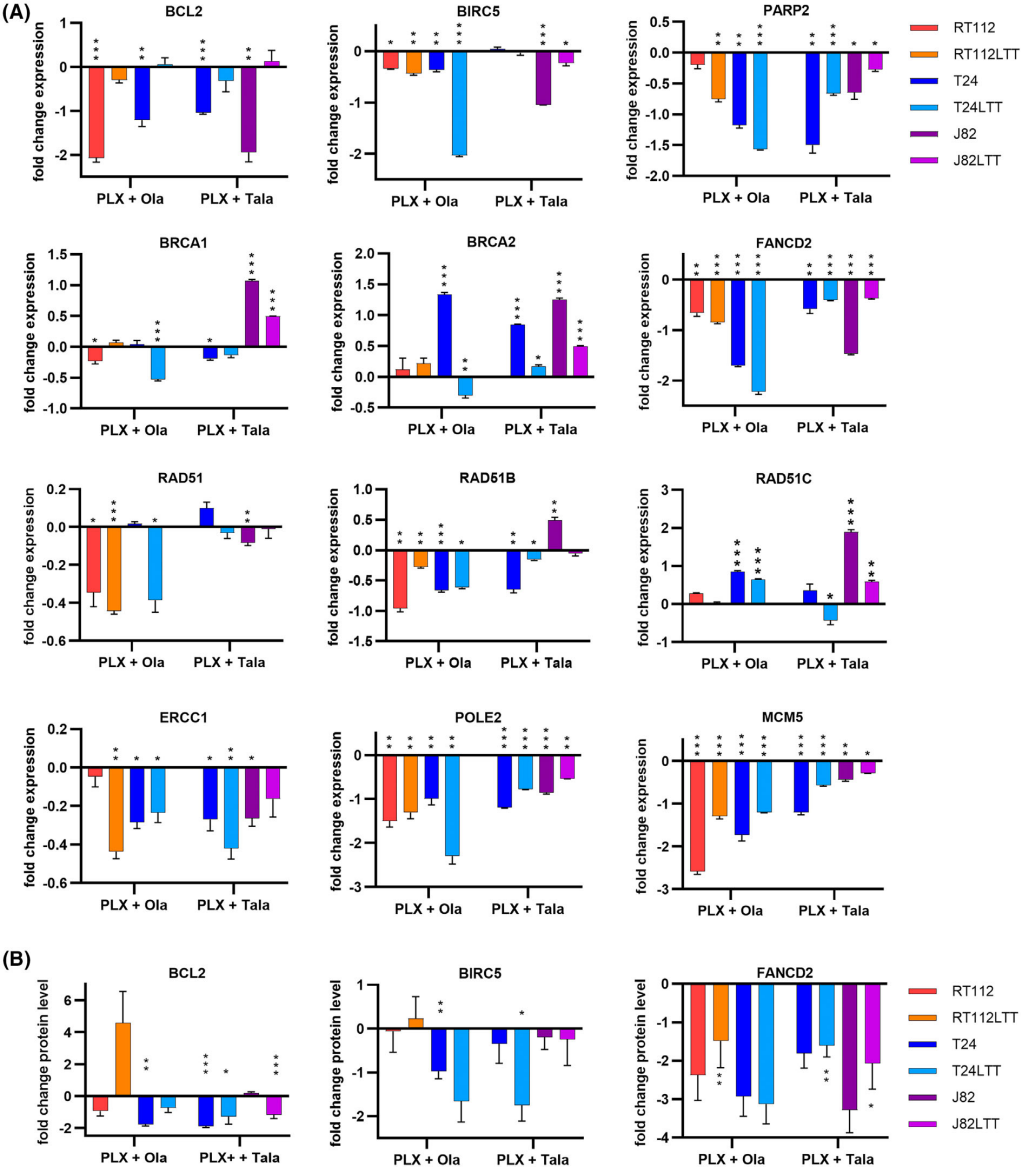
Validation of factors underlying synergism of combination treatment. (A) qRT‐PCR validation of genes after indicated treatment conditions with PLX51107 (PLX) and Olaparib (Ola) or Talazoparib (Tala) in indicated cell line pairs. *TBP* was measured as a housekeeping gene and used for normalization. Fold change expression (log_2_) was calculated vs. DMSO control (set to 0). Significance is denoted in comparison to DMSO ***P ≤ 0.001, **P ≤ 0.01, *P ≤ 0.05 (mean ± SEM; *n* = 3; two‐way ANOVA, Tukey's test). (B) Treatment‐induced changes of BCL2, BIRC5, and FANCD2 were further validated on the protein level by western blotting. Band intensities were quantified densitometrically using Image Lab Software (Bio‐Rad) and normalized to α‐Tubulin as loading control and DMSO as solvent control. Fold change expression (log_2_) was calculated vs. DMSO control (set to 0). Results of three independent experiments were averaged. Significance is denoted in comparison to DMSO, ***P ≤ 0.001, **P ≤ 0.01, *P ≤ 0.05 (mean ± SEM; *n* = 3; two‐way ANOVA, Tukey's test). Example western blot images are displayed in Fig. S7.

We also had previously identified factors underlying PLX response in UCC. We demonstrated that PLX mono‐treatment altered levels for regulators of cell cycle, apoptosis, and DNA damage response and induced a BRCAness‐like HRR‐deficient phenotype, so that induced DSB could be less efficiently repaired [20]. Thus, we measured whether altered expression of such factors might also contribute to the synergistic effects of the combination treatment. We found that anti‐apoptotic factors like *BCL2* and *BIRC5* (Survivin) were particularly downregulated in cell lines responding strongly to the combination treatment that were insensitive to a mono‐treatment like RT112 (poor responder to PARPi) and J82 (poor responder to BETi) (Fig. 6A). On the protein level, we found relevant reductions, too (Fig. 6B, Figs S7F, S8I,J). These results indicate that the reduction of anti‐apoptotic factors may underlie strong synergistic effects.

We further measured the expression of six genes involved in HRR. *FANCD2* was the only gene that was strongly downregulated across all cell lines by both combinations, thus contributing also mechanistically to treatment synergism. A very robust reduction could also be observed at the protein level (Fig. 6B, Fig. S7F). The other five HRR genes were not strongly reduced or rather induced by combination treatment (Fig. 6A).

Similarly, the DNA repair factors *PARP2*, *ERCC1* as well as *POLE2* and *MCM5* involved in DNA replication were downregulated in all cell lines (Fig. 6A). POLE2 and MCM5 are particularly interesting, as their downregulation by combination treatment contributes to blocked replication [44, 45] which renders cells sensitive to PARPi [46].

## 4 Discussion

In this study, we addressed the clinical need for additional UC‐specific biomarkers to increase PARPi treatment efficacy by more tailored treatment and for combination treatment approaches to increase efficacy. Clinical trials so far detected genomic alterations in HRR gene panels interrogated by clinically approved assays like Oncoplus or FoundationOne^®^ CDx as well as the expression of *SLFN11*. Alternatively, appropriate combination treatment may increase efficacy. Clinical trials for UC currently investigate combination approaches with newly approved UC therapies such as ICI or Enfortumab vedotin, an ADC. Olaparib, Rucaparib, Niraparib, and Talazoparib were used in clinical trials for UC, the first three also as a mono‐therapy. Talazoparib was studied in combination with ICI [14, 19]. In other solid cancers, particularly breast, ovarian, and prostate cancer, also combinations with, for example, anti‐angiogenic compounds or other kinase inhibitors are investigated [19].

We developed a new highly efficient PARPi‐based combination treatment for UC that is also potent after pretreatment with Cisplatin. Cellular responses of selected UC cell lines toward different PARPi as a mono‐treatment had been studied earlier. Bhattacharjee *et al*. [47] determined the effects of five different PARPi on proliferation and cell death induction of UMUC3, T24, and benign SV‐HUC1 cells. Rucaparib and Veliparib had high IC_50_ values in the intermediate micromolar range rendering them rather unsuitable. Niraparib and Talazoparib were the most effective compounds for single‐drug treatment *in vitro*. Simultaneous treatment with Cisplatin reduced cell survival *in vitro* and *in vivo*. In clinical trials, unselected Cisplatin‐pretreated patients did not respond significantly toward PARPi mono‐treatment [15, 19]. Other authors investigated the response of four Cisplatin‐naïve UC cell lines (UMUC3, T24, RT112, 5637) toward Olaparib and Talazoparib to combine with Palbociclib, a CDK4/6 inhibitor [48]. However, to our knowledge, Cisplatin‐pretreated UC cells have not been investigated for response toward PARPi *in vitro*, yet. Therefore, we investigated our own Cisplatin‐resistant cell models (LTTs) compared to their parental UC cell lines. We chose to compare the next‐generation, highly potent PARPi Talazoparib with the earlier approved PARPi Olaparib that is already widely studied in clinical trials. All LTT lines had lower IC_50_ values for Olaparib compared to their parentals. Such differences were not observed for Talazoparib, which was, however, generally more potent. RT112LTT and parental cells present an exception by being highly resistant toward both PARPi and thus suitable as a model to study PARPi resistance mechanisms in UC in order to identify new UC‐specific factors for PARPi response.

Toxicity to benign HBLAK cells was stronger for Ola than for Tala. In clinical trials for UC, the safety profile of PARPi was comparable with toxicities reported for other cancer types. Severe adverse events (AE) were rare [14]. However, in the BISCAY study, investigating the combination of Ola and the ICI Durvalumab, 40% of HRR‐selected patients stopped treatment due to Ola‐induced toxicity [49].

We found IC_50_ values of Olaparib much higher compared to Talazoparib in agreement with data by others [47, 48]. Differences in PARP trapping activity are discussed as one reason for this difference [7]. Notably, Talazoparib has the least off‐target effects on kinases, but Olaparib the most, which could also contribute to differences in efficacy, as well as altered gene expression [50] since PARP1 has also been suggested as a regulator of transcription [51]. However, since no data on effects of different PARPi on the transcriptome in UC are published in the GEO data repositorium of the NCBI, yet, we performed RNA sequencing of UC cells treated either with Ola or Tala. This analysis revealed huge differences in gene expression effects between the two PARPi and only a small number of genes commonly affected by both PARPi. Genes uniquely induced by Ola in T24 were enriched for innate immune responses, particularly antigen presentation, which could be advantageous for combined treatment with ICI. PD‐L1 was only induced by Tala in T24, but not in the other investigated UCC.

Among genes commonly regulated by both PARPi in T24, the 215 commonly induced genes were mainly enriched mainly for cell stress and immune response processes and included various cytokines and factors associated with defense to bacteria or viruses, especially cGAS‐STING and interferon signaling. Induction of cGAS‐STING signaling by Talazoparib was validated in this study. These factors may contribute to the beneficial effects of combining PARPi with ICI in UC patients [19]. One idea rationalizing the combination of PARPi with ICI is the induction of neoantigens improving recognition by T cells. Increased DNA damage could also result in the accumulation of cytosolic DNA thereby triggering cGAS‐STING signaling which also stimulates innate immune responses [52]. For breast and pancreatic cancer cells, induction of PD‐L1 expression by PARPi *in vitro* was reported [53, 54], but does not appear to occur regularly in UC according to our data. Published data suggest a relation between PD‐L1‐negative tumors and a BRCAness phenotype. Intriguingly, deletion of PD‐L1, but not its blockade by ICI, resulted in elevated DNA damage levels in BRCA1 wild‐type models and improved growth control by PARPi in some cell models, but not all cell models. Such results may explain why poor response toward PARPi cannot be generally improved by combination with ICI therapy *in vivo* [55], again highlighting the need for biomarkers predicting PARPi sensitivity prior to the application of such combination therapies. PD‐L1 may be taken into account as a marker since it is anyway recorded prior to ICI therapy, but needs further validation in large patient cohorts for use in combination therapy.

To analyze in more detail the molecular effect of the better PARPi Tala, we extended RNA Seq analysis to more UCC and LTT. Only a few genes, *CDKN1A*, *SAT1*, and *SLFN5*, were commonly induced by Talazoparib in all cell lines and may thus be UC‐specific Tala response markers. Some were not mentioned earlier in any PARP response context, yet. Induction of *CDKN1A*, encoding the cell cycle inhibitor p21^CIP1^, could belong to a general response toward replication stress and DNA damage and is likely to contribute to the observed G2 arrest. p21 induction by Olaparib has also been reported in U2OS and breast cancer cells [56, 57]. *SAT1* is a rate‐limiting enzyme for the acetylation of the polyamines spermine and spermidine, which regulate replication, translation, and chromatin condensation. SAT1 also functions in the transcriptional regulation of *BRCA1* and further regulators of DNA repair, cell cycle, and mitosis [58]. It was described as critical for the growth of glioma, elevated in high‐grade tumors, and associated with poor outcomes. Our analysis of TCGA data did not reveal a significant association with patient prognosis. There is one study on SAT1 function in UC suggesting relevance for Cisplatin resistance [59]. While SLFN11 is known to regulate replication stress and HRR, its paralog SLFN5 rather functions as a transcriptional corepressor in interferon signaling and could dampen the antineoplastic response of the immune system [60]. Only recently, a function of SLFN5 in DNA repair was reported, namely regulation of higher‐order chromatin topology in response to DNA damage. Its loss impairs NHEJ [61]. Since *SLFN5* was induced in all our cell lines in response to Tala, it might be part of a cellular attempt to increase NHEJ capacity for DNA repair. The only published study so far on the relation between *SLFN5* and PARPi reported that loss of *SLFN5* promoted PARPi resistance in BRCA1‐deficient U2OS cells [61]. Thus, lack of *SLFN5* and *SLFN11* seems to promote PARPi resistance, while—according to our data—their induction is associated with a favorable response to PARPi. Notably, *SLFN5* was frequently increased and correlated with worse prognosis in various cancers, for example, pancreatic ductal adenocarcinoma, glioma, colorectal adenocarcinoma, and gastric cancer [60, 62, 63]. Likewise, we found high levels of *SLFN5* to be correlated with shorter survival of UC patients within the TCGA cohort. SLFN5 was also found associated with immune responses affecting immune checkpoints, immune cell infiltration and may thus be involved in tumor immune evasion [63, 64]. Further findings with regard to solid cancer types have recently been reviewed [65], but SLFN5 has not been studied in UC, yet. We report here for the first time on a potential functional relevance of SLFN5 for patient prognosis and PARPi response in UC, also with regard to combined treatment with immunotherapies in clinical trials.

Seeking to identify treatment response biomarkers for PARPi in UC, our results suggest that patient stratification by HRR gene status only may not be sufficient for UC patients and inclusion of further biomarkers may be required. According to our analyses, PARPi resistance factors (shieldins, etc.) discussed in the literature appeared not to be important in UC, except for SLFN11. SLFN11 is recruited during replication stress to induce an irreversible replication block, and its loss is associated with PARPi resistance in other cancer types [46]. Since we found PARPi‐resistant RT112 not to display any SLFN11 protein, but also not to carry genetic alterations of *SLFN11*, our data revealed that also loss of expression by epigenetic regulation instead of genetic loss may contribute to PARPi resistance. Expression loss can be epigenetically regulated, for example, by DNA hypermethylation of its promoter [66]. DNA methylation alterations as a mechanism of PARPi resistance have been studied very recently in ovarian cancer [67]. *SLFN11* relevance for PARPi resistance could be functionally validated in the present study by siRNA knockdown. Further validation of PARPi response prediction is needed in large patient cohorts. Other discussed resistance factors are loss of *p53BP1* and *SHLD1/2* of the shieldin complex, for example, in breast cancer [27], which occurs rarely in UC according to analyses. Likewise, *PARP1* and *PARG* mutations suggested as resistance mechanisms [68] also do not apply to UC.

Instead, we identified OAS1 as a UC‐specific predictor for PARPi response that also correlated with patient survival. While high *OAS1* expression correlated with PARPi resistance, expression was strongly reduced by our combination treatments and may contribute to synergism. *OAS1* belongs to the interferon‐stimulated genes (ISG) and binds transcription factor IRF1 to enhance the innate immune response and restrict viral replication [69]. Physiologically, ISGs are associated with cGAS‐STING signaling in the response to cytosolic DNA that usually originates from infections but also from treatments damaging DNA, like our combination treatment. Upregulation of ISGs is known to contribute to resistance against DNA‐damaging therapies [70]. A pan‐cancer analysis of public data for *OAS1* revealed, however, differences between cancer types and various functions. Expression in UC was in general increased which could contribute to poor response toward PARPi mono‐treatment in some clinical trials. Concurringly, we observed a slight reduction of IC_50_ values after siRNA knockdown of OAS1. *OAS1* upregulation was also reported to diminish the benefits of tumor‐infiltrating lymphocytes on patient survival and to contribute to immunotherapy resistance [71]. A relation with immune infiltration was confirmed by other *in silico* analyses of lung and bladder cancer data [72, 73]. Reduction of *OAS1* expression by our combination treatment would therefore be favorable for immunotherapy. We observed an inverse relationship between *OAS1* and *SLFN11*, so that both markers should be investigated together in future clinical trials.

Apart from biomarker discovery, the development of synergizing combination therapies is another approach to increase the efficacy of PARPi. Among epigenetic inhibitors, mainly the combination of PARPi with DNA methylation inhibitors has been investigated in clinical trials [51]. *In vitro*, other combinations with HDACi [74] or BETi, particularly the first‐generation BETi JQ1 and Olaparib, have been studied in cell lines from breast, ovarian, and prostate cancer [75]. Synergism with Talazoparib was found for JQ1 in multiple cancer cell lines of different origin regardless of BRCA status and in *in vivo* xenograft models, but has not been investigated for UC [76]. These authors also generated PARPi‐resistant cell models that could be sensitized again by BET inhibition. Together with our results on UC, such data indicate that clinical issues with poor response toward PARPi mono‐treatment can be overcome by combination with a BETi in the majority of cases and according to our data also in the setting following Cisplatin pretreatment.

From our previous analyses, we knew that our benign control cells were sensitive to the first generation BETi JQ1 [77]. Therefore, we investigated PLX51107 as a new BETi considered having better toxicity profiles [25]. We had previously characterized the cellular and molecular effect of PLX mono‐treatment on benign controls and UCC that could sensitize to PARPi [20]. The results of our current study demonstrate that the BETi PLX indeed synergizes with both investigated PARPi, but particularly strong with Tala. Synergism was achieved in UCC and also in LTTs that were regularly pretreated with Cisplatin and likewise in respective bladder cancer PDO models. We found that strong synergistic effects allowed dose reduction of both compounds which still induced cell death in cancer cells but affected diverse benign control cells less. We demonstrated that combination treatment induced DNA damage, which could not be regularly repaired since we observed a reduction of discrete RAD51 repair foci in the vicinity of ɣH2AX. Since HRR activity was shown to correlate with RAD51 foci formation [9, 78], it appears to be impaired by the combination. Our molecular analyses revealed that the expression of some HRR genes was reduced by treatment. Reduction of HRR genes by different BETi (JQ1 and GSK525762A) has been reported, for example, in breast and ovarian cancer cells sensitizing to Olaparib [79, 80]. We found *FANCD2* to be the most strongly downregulated gene by both combinations in all UCC. Since BRCA2 and FANCD2 colocalize at DNA damage sites and cells with *FANCD2* mutations display reduced HRR rates [81], we conclude its strong downregulation by both combinations contributes to observed HRR deficiency and underlies treatment synergism. FANCD2 functions in response to replication stress may also be relevant [82]; it interacts with MCM replicative helicases that further recruit RPA and PCNA [46]. Our combination treatment also significantly reduced *MCM5* and *POLE2* suggesting also impact on DNA replication. Further, we found downregulation of anti‐apoptotic factors like to contribute to synergism of combination treatment.

## 5 Conclusions

With our study, we suggest new predictive biomarker candidates SLFN5, SLFN11, and OAS1 for PARPi response in UC that need further validation in cohorts of clinical trials. With the combination of the BETi PLX51107 and the PARPi Talazoparib, we developed a new highly efficient combination treatment for targeted therapy of UC. Treatment synergism allows dose reduction thus limiting toxicity to normal cells. The novel combination treatment was efficient in all investigated cell models, including otherwise PARPi‐resistant and Cisplatin‐pretreated cells; the latter is a frequent setting in UC patients. Thus, this combination treatment would be suitable as an all‐comer approach and may alleviate the need for biomarkers for prediction of response to PARPi.

## Conflict of interest

GN has served on advisory boards for and received honoraria as a lecturer as well as travel expenses and congress registration costs from Astellas Pharma and Pfizer Inc. The other authors declare that they have no further competing interests.

## Author contributions

Conceptualization and design, MJH and GN; data Curation, JS, MJH, PP, KK, MS, TCMZ, and TM; formal analysis and data interpretation, JS, AB, NK, PP, MR, LL, JV, AS, BF, VS, and MJH; funding acquisition, GN and MJH; investigation and data acquisition, JS, AB, NK, PP, MR, LL, JV, AS, VS, BF, and MJH; methodology, MJH, PP, MR, KK, MS, TM, and GN; project administration, MJH and GN; resources, MJH, GN, KK, MS, TCMZ, and TM; software, JS, MJH, PP, and MR; supervision, MJH and GN; validation, JS, AB, PP, MR, LL, JV, AS, BF, VS, and MJH; visualization, JS and MJH; writing—original draft, MJH, JS, and GN; writing—review and editing, JS, MJH, GN, MR, AB, PP, LL, JV, KK, NK, VS, AS, BF, MS, TM, and TCMZ. All authors have read and agreed to the published version of the manuscript.

## Ethics statement

The study involving human blood donors was approved by the Ethics Committee of the University Hospital Duesseldorf (2023‐2667; 2025‐3381). Organoid cultures were generated at Erasmus University Medical Center. The study was approved by the Institutional Review Board (IRB) of the Erasmus University Medical Center (MEC‐2021‐0354). Written informed consent to participate was provided by the participants. Performed procedures were in accordance with the Declaration of Helsinki.

## Supporting information


**Fig. S1.** Additional IC_50_ values, PROTAC efficiency, selection of reduced dosages and normal toxicity of combination treatment.

**Fig. S2.** Additional analyses for normal toxicity.

**Fig. S3.** Genetic alterations in homologous recombination repair genes are frequent in breast cancer cell lines.

**Fig. S4.** Genetic alterations in homologous recombination repair genes are less frequent in urothelial carcinoma.

**Fig. S5.** Correlation analysis for homologous recombination repair gene expression and PARP inhibitor response.

**Fig. S6.** Alterations in putative PARP inhibitor resistance factors in urothelial carcinoma.

**Fig. S7.** Further validation data for *OAS1*, cGAS‐STING and synergism factors.

**Fig. S8.** Raw western blot membranes.

**Table S1.** Information on primer and qRT‐PCR protocols.

**Table S2.** Reduced dosages applied in experiments are given in detail.

**Table S3.** Genes commonly regulated by the two PARP inhibitors Olaparib and Talazoparib in the T24 cell line pair.

**Table S4.** Genes uniquely regulated by either PARP inhibitor Olaparib or Talazoparib in the T24 cell line pair.

**Table S5.** Genes commonly regulated by the PARP inhibitor Talazoparib in all investigated cell lines and genes only regulated by Talazoparib in RT112 cell line.

## Data Availability

The RNA Seq datasets generated and analyzed during the current study are available in the Gene Expression Omnibus (GEO) repository (NCBI; GSE285648). Public data were assessed using the above indicated websites or online tools. All data generated or analyzed during this study are included in this published article and its supplementary information files.

## References

[1] Ferlay J , Ervik M , Lam F , Laversanne M , Colombet M , Mery L , et al. Global Cancer Observatory: Cancer Today. Lyon, France: International Agency for Research on Cancer; 2024. https://gco.iarc.who.int/today

[2] Meeks JJ , Black PC , Galsky M , Grivas P , Hahn NM , Hussain SA , et al. Checkpoint inhibitors in urothelial carcinoma—future directions and biomarker selection. Eur Urol. 2023;84:473–483. 10.1016/j.eururo.2023.05.011 37258363

[3] Klümper N , Tran NK , Zschäbitz S , Hahn O , Büttner T , Roghmann F , et al. NECTIN4 amplification is frequent in solid tumors and predicts Enfortumab Vedotin response in metastatic urothelial cancer. J Clin Oncol. 2024;42:2446–2455. 10.1200/jco.23.01983 38657187 PMC11227306

[4] Robertson AG , Kim J , Al‐Ahmadie H , Bellmunt J , Guo G , Cherniack AD , et al. Comprehensive molecular characterization of muscle‐invasive bladder cancer. Cell. 2017;171:540–556. 10.1016/j.cell.2017.09.007 28988769 PMC5687509

[5] Helleday T . The underlying mechanism for the PARP and BRCA synthetic lethality: clearing up the misunderstandings. Mol Oncol. 2011;5:387–393. 10.1016/j.molonc.2011.07.001 21821475 PMC5528309

[6] Lord CJ , Ashworth A . PARP inhibitors: synthetic lethality in the clinic. Science. 2017;355:1152–1158. 10.1126/science.aam7344 28302823 PMC6175050

[7] Murai J , Pommier Y . Classification of PARP inhibitors based on PARP trapping and catalytic inhibition, and rationale for combinations with topoisomerase I inhibitors and alkylating agents. In: Curtin NJ , Sharma RA , editors. PARP inhibitors for cancer therapy. Cham: Springer International Publishing; 2015. p. 261–274.

[8] Pommier Y , O'Connor MJ , de Bono J . Laying a trap to kill cancer cells: PARP inhibitors and their mechanisms of action. Sci Transl Med. 2016;8:362ps317. 10.1126/scitranslmed.aaf9246 27797957

[9] Longoria O , Beije N , de Bono JS . PARP inhibitors for prostate cancer. Semin Oncol. 2024;51:25–35. 10.1053/j.seminoncol.2023.09.003 37783649

[10] Wang SSY , Jie YE , Cheng SW , Ling GL , Ming HVY . PARP inhibitors in breast and ovarian cancer. Cancers. 2023;15:2357. 10.3390/cancers15082357 37190285 PMC10137187

[11] Chandran E , Simon NI , Niglio SA , Ley L , Cordes LM , Banday R , et al. A phase II study of Olaparib (AZD2281) in patients with metastatic/advanced urothelial carcinoma and other genitourinary tumors with DNA‐repair defects. 2017.

[12] Agarwal N , Azad AA , Carles J , Fay AP , Matsubara N , Heinrich D , et al. Talazoparib plus enzalutamide in men with first‐line metastatic castration‐resistant prostate cancer (TALAPRO‐2): a randomised, placebo‐controlled, phase 3 trial. Lancet. 2023;402:291–303. 10.1016/s0140-6736(23)01055-3 37285865

[13] Mekonnen N , Yang H , Shin YK . Homologous recombination deficiency in ovarian, breast, colorectal, pancreatic, non‐small cell lung and prostate cancers, and the mechanisms of resistance to PARP inhibitors. Front Oncol. 2022;12:880643. 10.3389/fonc.2022.880643 35785170 PMC9247200

[14] Crabb SJ , Khalid T , Woods L , Frampton G , Shepherd J . PARP inhibitors for metastatic urothelial carcinoma: a systematic review of efficacy and safety. Bladder Cancer. 2023;9:365–376. 10.3233/blc-230071 38994249 PMC11165942

[15] Gamba T , Paparo J , Panepinto O , Dionisio R , Di Maio M , Vignani F . Poly (ADP‐ribose) polymerase inhibitors in patients with urothelial cancer. Clin Genitourin Cancer. 2023;21:509–516. 10.1016/j.clgc.2023.07.009 37500375

[16] Fulton B , Jones R , Powles T , Crabb S , Paul J , Birtle A , et al. ATLANTIS: a randomised multi‐arm phase II biomarker‐directed umbrella screening trial of maintenance targeted therapy after chemotherapy in patients with advanced or metastatic urothelial cancer. Trials. 2020;21:344. 10.1186/s13063-020-04283-5 32306987 PMC7168999

[17] Rosenberg JE , Park SH , Kozlov V , Dao TV , Castellano D , Li JR , et al. Durvalumab plus Olaparib in previously untreated, platinum‐ineligible patients with metastatic urothelial carcinoma: a multicenter, randomized, phase II trial (BAYOU). J Clin Oncol. 2023;41:43–53. 10.1200/jco.22.00205 35737919 PMC9788981

[18] Vignani F , Tambaro R , De Giorgi U , Giannatempo P , Bimbatti D , Carella C , et al. Addition of Niraparib to best supportive care as maintenance treatment in patients with advanced urothelial carcinoma whose disease did not Progress after first‐line platinum‐based chemotherapy: the meet‐URO12 randomized phase 2 trial. Eur Urol. 2023;83:82–89. 10.1016/j.eururo.2022.09.025 36216658

[19] D'Andrea VD , Magnani CJ , Ernandez J , Bellmunt J , Mossanen M , Clinton TN , et al. Impact of DNA repair deficiency in the evolving treatment landscape of bladder cancer. Curr Urol Rep. 2024;26:12. 10.1007/s11934-024-01242-4 39382743

[20] Thy S , Hommel A , Meneceur S , Bartkowiak AL , Schulz WA , Niegisch G , et al. Epigenetic treatment of urothelial carcinoma cells sensitizes to cisplatin chemotherapy and PARP inhibitor treatment. Cancers. 2021;13:1376. 10.3390/cancers13061376 33803654 PMC8002916

[21] Ferri E , Petosa C , McKenna CE . Bromodomains: structure, function and pharmacology of inhibition. Biochem Pharmacol. 2016;106:1–18. 10.1016/j.bcp.2015.12.005 26707800

[22] Yang Z , Yik JH , Chen R , He N , Jang MK , Ozato K , et al. Recruitment of P‐TEFb for stimulation of transcriptional elongation by the bromodomain protein Brd4. Mol Cell. 2005;19:535–545. 10.1016/j.molcel.2005.06.029 16109377

[23] Wu X , Liu D , Tao D , Xiang W , Xiao X , Wang M , et al. BRD4 regulates EZH2 transcription through upregulation of C‐MYC and represents a novel therapeutic target in bladder cancer. Mol Cancer Ther. 2016;15:1029–1042. 10.1158/1535-7163.Mct-15-0750 26939702

[24] Yan Y , Yang FQ , Zhang HM , Li J , Li W , Wang GC , et al. Bromodomain 4 protein is a predictor of survival for urothelial carcinoma of bladder. Int J Clin Exp Pathol. 2014;7:4231–4238. 25120803 PMC4129038

[25] Ozer HG , El‐Gamal D , Powell B , Hing ZA , Blachly JS , Harrington B , et al. BRD4 profiling identifies critical chronic lymphocytic leukemia oncogenic circuits and reveals sensitivity to PLX51107, a novel structurally distinct BET inhibitor. Cancer Discov. 2018;8:458–477. 10.1158/2159-8290.Cd-17-0902 29386193 PMC5882533

[26] Skowron MA , Melnikova M , Van Roermund JGH , Romano A , Albers P , Thomale J , et al. Multifaceted mechanisms of cisplatin resistance in long‐term treated urothelial carcinoma cell lines. Int J Mol Sci. 2018;19:590. 29462944 10.3390/ijms19020590 PMC5855812

[27] Bhamidipati D , Haro‐Silerio JI , Yap TA , Ngoi N . PARP inhibitors: enhancing efficacy through rational combinations. Br J Cancer. 2023;129:904–916. 10.1038/s41416-023-02326-7 37430137 PMC10491787

[28] Noordermeer SM , van Attikum H . PARP inhibitor resistance: a tug‐of‐war in BRCA‐mutated cells. Trends Cell Biol. 2019;29:820–834. 10.1016/j.tcb.2019.07.008 31421928

[29] Hoffmann MJK , Skowron MA , Pinkerneil M , Niegisch G , Brandt A , et al. The new immortalized Uroepithelial cell line HBLAK contains defined genetic aberrations typical of early stage urothelial tumors. Bladder Cancer. 2016;2:449–463. 28035326 10.3233/BLC-160065 PMC5181672

[30] Mullenders J , de Jongh E , Brousali A , Roosen M , Blom JPA , Begthel H , et al. Mouse and human urothelial cancer organoids: a tool for bladder cancer research. Proc Natl Acad Sci USA. 2019;116:4567–4574. 10.1073/pnas.1803595116 30787188 PMC6410883

[31] Scholtes MP , Akbarzadeh M , Galaras A , Nakauma‐Gonzáles JA , Bazrafshan A , Solanki V , et al. Integrative analysis of patient‐derived tumoroids and ex vivo organoid modelling of ARID1A loss in bladder cancer reveals therapeutic molecular targets. Cancer Lett. 2025;614:217506. 10.1016/j.canlet.2025.217506 39892702

[32] Meneceur S , Grunewald CM , Niegisch G , Hoffmann MJ . Epigenetic priming and development of new combination therapy approaches. Urothelial carcinoma: methods and protocols. New York, NY: Springer; 2023. p. 259–281. 10.1007/978-1-0716-3291-8_16 37410240

[33] Chou TC . Drug combination studies and their synergy quantification using the Chou‐Talalay method. Cancer Res. 2010;70:440–446. 20068163 10.1158/0008-5472.CAN-09-1947

[34] Di Veroli GY , Fornari C , Wang D , Mollard S , Bramhall JL , Richards FM , et al. Combenefit: an interactive platform for the analysis and visualization of drug combinations. Bioinformatics. 2016;32:2866–2868. 10.1093/bioinformatics/btw230 27153664 PMC5018366

[35] Pinkerneil MH , Deenen R , Köhrer K , Arent T , Schulz WA , et al. Inhibition of class I histone deacetylases 1 and 2 promotes urothelial carcinoma cell death by various mechanisms. Mol Cancer Ther. 2016;15:299–312. 26772204 10.1158/1535-7163.MCT-15-0618

[36] Lehmann M , Hoffmann MJ , Koch A , Ulrich SM , Schulz WA , Niegisch G . Histone deacetylase 8 is deregulated in urothelial cancer but not a target for efficient treatment. J Exp Clin Cancer Res. 2014;33:59. 10.1186/s13046-014-0059-8 25011684 PMC4230422

[37] Pinkerneil MH , Niegisch G . Epigenetic treatment options in urothelial carcinoma. Methods Mol Biol. 2018;1655:289–317. 28889393 10.1007/978-1-4939-7234-0_21

[38] Lang A , Yilmaz M , Hader C , Murday S , Kunz X , Wagner N , et al. Contingencies of UTX/KDM6A action in urothelial carcinoma. Cancers. 2019;11:481. 10.3390/cancers11040481 30987376 PMC6520694

[39] Oliveros JC . An interactive tool for comparing lists with Venn's diagrams, Venny. 2007‐2015.

[40] Huang D , Sherman BT , Lempicki RA . Systematic and integrative analysis of large gene lists using DAVID bioinformatics resources. Nat Protoc. 2009;4:44–57. 10.1038/nprot.2008.211 19131956

[41] Network TCGAR . Comprehensive molecular characterization of urothelial bladder carcinoma. Nature. 2014;507:315–322. 10.1038/nature12965 24476821 PMC3962515

[42] Cerami E , Gao J , Dogrusoz U , Gross BE , Sumer SO , Aksoy BA , et al. The cBio cancer genomics portal: an open platform for exploring multidimensional cancer genomics data. Cancer Discov. 2012;2:401–404. 10.1158/2159-8290.Cd-12-0095 22588877 PMC3956037

[43] Gao J , Aksoy BA , Dogrusoz U , Dresdner G , Gross B , Sumer SO , et al. Integrative analysis of complex cancer genomics and clinical profiles using the cBioPortal. Sci Signal. 2013;6:pl1. 10.1126/scisignal.2004088 23550210 PMC4160307

[44] Rogers RF , Walton MI , Cherry DL , Collins I , Clarke PA , Garrett MD , et al. CHK1 inhibition is synthetically lethal with loss of B‐family DNA polymerase function in human lung and colorectal cancer cells. Cancer Res. 2020;80:1735–1747. 10.1158/0008-5472.Can-19-1372 32161100 PMC7611445

[45] Stoeber K , Tlsty TD , Happerfield L , Thomas GA , Romanov S , Bobrow L , et al. DNA replication licensing and human cell proliferation. J Cell Sci. 2001;114:2027–2041. 10.1242/jcs.114.11.2027 11493639

[46] Murai J , Tang SW , Leo E , Baechler SA , Redon CE , Zhang H , et al. SLFN11 blocks stressed replication forks independently of ATR. Mol Cell. 2018;69:371–384. 10.1016/j.molcel.2018.01.012 29395061 PMC5802881

[47] Bhattacharjee S , Sullivan MJ , Wynn RR , Demagall A , Hendrix AS , Sindhwani P , et al. PARP inhibitors chemopotentiate and synergize with cisplatin to inhibit bladder cancer cell survival and tumor growth. BMC Cancer. 2022;22:312. 10.1186/s12885-022-09376-9 35321693 PMC8944004

[48] Klein FG , Granier C , Zhao Y , Pan Q , Tong Z , Gschwend JE , et al. Combination of Talazoparib and Palbociclib as a potent treatment strategy in bladder cancer. J Pers Med. 2021;11:340. 10.3390/jpm11050340 33923231 PMC8145096

[49] Powles T , Carroll D , Chowdhury S , Gravis G , Joly F , Carles J , et al. An adaptive, biomarker‐directed platform study of durvalumab in combination with targeted therapies in advanced urothelial cancer. Nat Med. 2021;27:793–801. 10.1038/s41591-021-01317-6 33941921

[50] Kim DS , Camacho CV , Kraus WL . Alternate therapeutic pathways for PARP inhibitors and potential mechanisms of resistance. Exp Mol Med. 2021;53:42–51. 10.1038/s12276-021-00557-3 33487630 PMC8080675

[51] Rose M , Burgess JT , O'Byrne K , Richard DJ , Bolderson E . PARP inhibitors: clinical relevance, mechanisms of action and tumor resistance. Front Cell Dev Biol. 2020;8:564601. 10.3389/fcell.2020.564601 33015058 PMC7509090

[52] Prasanna T , Wu F , Khanna KK , Yip D , Malik L , Dahlstrom JE , et al. Optimizing poly (ADP‐ribose) polymerase inhibition through combined epigenetic and immunotherapy. Cancer Sci. 2018;109:3383–3392. 10.1111/cas.13799 30230653 PMC6215877

[53] Jiao S , Xia W , Yamaguchi H , Wei Y , Chen MK , Hsu JM , et al. PARP inhibitor upregulates PD‐L1 expression and enhances cancer‐associated immunosuppression. Clin Cancer Res. 2017;23:3711–3720. 10.1158/1078-0432.Ccr-16-3215 28167507 PMC5511572

[54] Wang Y , Zheng K , Xiong H , Huang Y , Chen X , Zhou Y , et al. PARP inhibitor upregulates PD‐L1 expression and provides a new combination therapy in pancreatic cancer. Front Immunol. 2021;12:762989. 10.3389/fimmu.2021.762989 34975854 PMC8718453

[55] Kornepati AVR , Boyd JT , Murray CE , Saifetiarova J , de la Peña Avalos B , Rogers CM , et al. Tumor intrinsic PD‐L1 promotes DNA repair in distinct cancers and suppresses PARP inhibitor‐induced synthetic lethality. Cancer Res. 2022;82:2156–2170. 10.1158/0008-5472.Can-21-2076 35247877 PMC9987177

[56] Jelinic P , Levine DA . New insights into PARP inhibitors' effect on cell cycle and homology‐directed DNA damage repair. Mol Cancer Ther. 2014;13:1645–1654. 10.1158/1535-7163.Mct-13-0906-t 24694947

[57] Zonneville J , Wang M , Alruwaili MM , Smith B , Melnick M , Eng KH , et al. Selective therapeutic strategy for p53‐deficient cancer by targeting dysregulation in DNA repair. Commun Biol. 2021;4:862. 10.1038/s42003-021-02370-0 34253820 PMC8275734

[58] Thakur VS , Aguila B , Brett‐Morris A , Creighton CJ , Welford SM . Spermidine/spermine N1‐acetyltransferase 1 is a gene‐specific transcriptional regulator that drives brain tumor aggressiveness. Oncogene. 2019;38:6794–6800. 10.1038/s41388-019-0917-0 31399646 PMC6786946

[59] Yeon A , You S , Kim M , Gupta A , Park MH , Weisenberger DJ , et al. Rewiring of cisplatin‐resistant bladder cancer cells through epigenetic regulation of genes involved in amino acid metabolism. Theranostics. 2018;8:4520–4534. 10.7150/thno.25130 30214636 PMC6134931

[60] Arslan AD , Sassano A , Saleiro D , Lisowski P , Kosciuczuk EM , Fischietti M , et al. Human SLFN5 is a transcriptional co‐repressor of STAT1‐mediated interferon responses and promotes the malignant phenotype in glioblastoma. Oncogene. 2017;36:6006–6019. 10.1038/onc.2017.205 28671669 PMC5821504

[61] Huang J , Wu C , Kloeber JA , Gao H , Gao M , Zhu Q , et al. SLFN5‐mediated chromatin dynamics sculpt higher‐order DNA repair topology. Mol Cell. 2023;83:1043–1060. 10.1016/j.molcel.2023.02.004 36854302 PMC10467573

[62] Fischietti M , Eckerdt F , Blyth GT , Arslan AD , Mati WM , Oku CV , et al. Schlafen 5 as a novel therapeutic target in pancreatic ductal adenocarcinoma. Oncogene. 2021;40:3273–3286. 10.1038/s41388-021-01761-1 33846574 PMC8106654

[63] Wu YJ , Chiao CC , Chuang PK , Hsieh CB , Ko CY , Ko CC , et al. Comprehensive analysis of bulk and single‐cell RNA sequencing data reveals Schlafen‐5 (SLFN5) as a novel prognosis and immunity. Int J Med Sci. 2024;21:2348–2364. 10.7150/ijms.97975 39310264 PMC11413889

[64] Xu J , Chen S , Liang J , Hao T , Wang H , Liu G , et al. Schlafen family is a prognostic biomarker and corresponds with immune infiltration in gastric cancer. Front Immunol. 2022;13:922138. 10.3389/fimmu.2022.922138 36090985 PMC9452737

[65] Tu T , Yuan Y , Liu X , Liang X , Yang X , Yang Y . Progress in investigating the relationship between Schlafen5 genes and malignant tumors. Front Oncol. 2023;13:1248825. 10.3389/fonc.2023.1248825 37771431 PMC10523568

[66] Nogales V , Reinhold WC , Varma S , Martinez‐Cardus A , Moutinho C , Moran S , et al. Epigenetic inactivation of the putative DNA/RNA helicase SLFN11 in human cancer confers resistance to platinum drugs. Oncotarget. 2016;7:3084–3097. 10.18632/oncotarget.6413 26625211 PMC4823092

[67] Senturk Kirmizitas T , van den Berg C , Boers R , Helmijr J , Makrodimitris S , Dag HH , et al. Epigenetic and genomic hallmarks of PARP‐inhibitor resistance in ovarian cancer patients. Genes. 2024;15:60750. 10.3390/genes15060750 PMC11203368 38927686

[68] Gogola E , Duarte AA , de Ruiter JR , Wiegant WW , Schmid JA , de Bruijn R , et al. Selective loss of PARG restores PARylation and counteracts PARP inhibitor‐mediated synthetic lethality. Cancer Cell. 2018;33:1078–1093. 10.1016/j.ccell.2018.05.008 29894693

[69] Harioudh MK , Perez J , So L , Maheshwari M , Ebert TS , Hornung V , et al. The canonical antiviral protein oligoadenylate synthetase 1 elicits antibacterial functions by enhancing IRF1 translation. Immunity. 2024;57:1812–1827. 10.1016/j.immuni.2024.06.003 38955184 PMC11324410

[70] Erdal E , Haider S , Rehwinkel J , Harris AL , McHugh PJ . A prosurvival DNA damage‐induced cytoplasmic interferon response is mediated by end resection factors and is limited by Trex1. Genes Dev. 2017;31:353–369. 10.1101/gad.289769.116 28279982 PMC5358756

[71] Yang R , Du Y , Zhang M , Liu Y , Feng H , Liu R , et al. Multi‐omics analysis reveals interferon‐stimulated gene OAS1 as a prognostic and immunological biomarker in pan‐cancer. Front Immunol. 2023;14:1249731. 10.3389/fimmu.2023.1249731 37928544 PMC10623006

[72] Luo D , Fang M , Shao L , Wang J , Liang Y , Chen M , et al. The EMT‐related genes GALNT3 and OAS1 are associated with immune cell infiltration and poor prognosis in lung adenocarcinoma. Front Biosci. 2023;28:271. 10.31083/j.fbl2810271 37919050

[73] Yue SY , Niu D , Liu XH , Li WY , Ding K , Fang HY , et al. BLCA prognostic model creation and validation based on immune gene‐metabolic gene combination. Discov Oncol. 2023;14:232. 10.1007/s12672-023-00853-6 38103068 PMC10725402

[74] Khalid U , Simovic M , Hammann LA , Iskar M , Wong JKL , Kumar R , et al. A synergistic interaction between HDAC‐ and PARP inhibitors in childhood tumors with chromothripsis. Int J Cancer. 2022;151:590–606. 10.1002/ijc.34027 35411591

[75] Yang L , Zhang Y , Shan W , Hu Z , Yuan J , Pi J , et al. Repression of BET activity sensitizes homologous recombination‐proficient cancers to PARP inhibition. Sci Transl Med. 2017;9:1645. 10.1126/scitranslmed.aal1645 PMC5705017 28747513

[76] Sun C , Yin J , Fang Y , Chen J , Jeong KJ , Chen X , et al. BRD4 inhibition is synthetic lethal with PARP inhibitors through the induction of homologous recombination deficiency. Cancer Cell. 2018;33:401–416. 10.1016/j.ccell.2018.01.019 29533782 PMC5944839

[77] Hölscher AS , Schulz WA , Pinkerneil M , Niegisch G , Hoffmann MJ . Combined inhibition of BET proteins and class I HDACs synergistically induces apoptosis in urothelial carcinoma cell lines. Clin Epigenetics. 2018;10:1. 10.1186/s13148-017-0434-3 29312470 PMC5755363

[78] Cruz C , Castroviejo‐Bermejo M , Gutiérrez‐Enríquez S , Llop‐Guevara A , Ibrahim YH , Gris‐Oliver A , et al. RAD51 foci as a functional biomarker of homologous recombination repair and PARP inhibitor resistance in germline BRCA‐mutated breast cancer. Ann Oncol. 2018;29:1203–1210. 10.1093/annonc/mdy099 29635390 PMC5961353

[79] Mio C , Gerratana L , Bolis M , Caponnetto F , Zanello A , Barbina M , et al. BET proteins regulate homologous recombination‐mediated DNA repair: BRCAness and implications for cancer therapy. Int J Cancer. 2019;144:755–766. 10.1002/ijc.31898 30259975

[80] Wilson AJ , Stubbs M , Liu P , Ruggeri B , Khabele D . The BET inhibitor INCB054329 reduces homologous recombination efficiency and augments PARP inhibitor activity in ovarian cancer. Gynecol Oncol. 2018;149:575–584. 10.1016/j.ygyno.2018.03.049 29567272 PMC5986599

[81] Moldovan GL , D'Andrea AD . How the fanconi anemia pathway guards the genome. Annu Rev Genet. 2009;43:223–249. 10.1146/annurev-genet-102108-134222 19686080 PMC2830711

[82] Kais Z , Rondinelli B , Holmes A , O'Leary C , Kozono D , D'Andrea AD , et al. FANCD2 maintains fork stability in BRCA1/2‐deficient tumors and promotes alternative end‐joining DNA repair. Cell Rep. 2016;15:2488–2499. 10.1016/j.celrep.2016.05.031 27264184 PMC4939765

