# Supplementary figures and images for: Improving PARP inhibitor efficacy in bladder cancer without genetic BRCAness by combination with PLX51107
Source: Mol Oncol. 2025 Nov 11;20(3):779–803. doi: 10.1002/1878-0261.70148 (PMC13042512; doi:10.1002/1878-0261.70148)

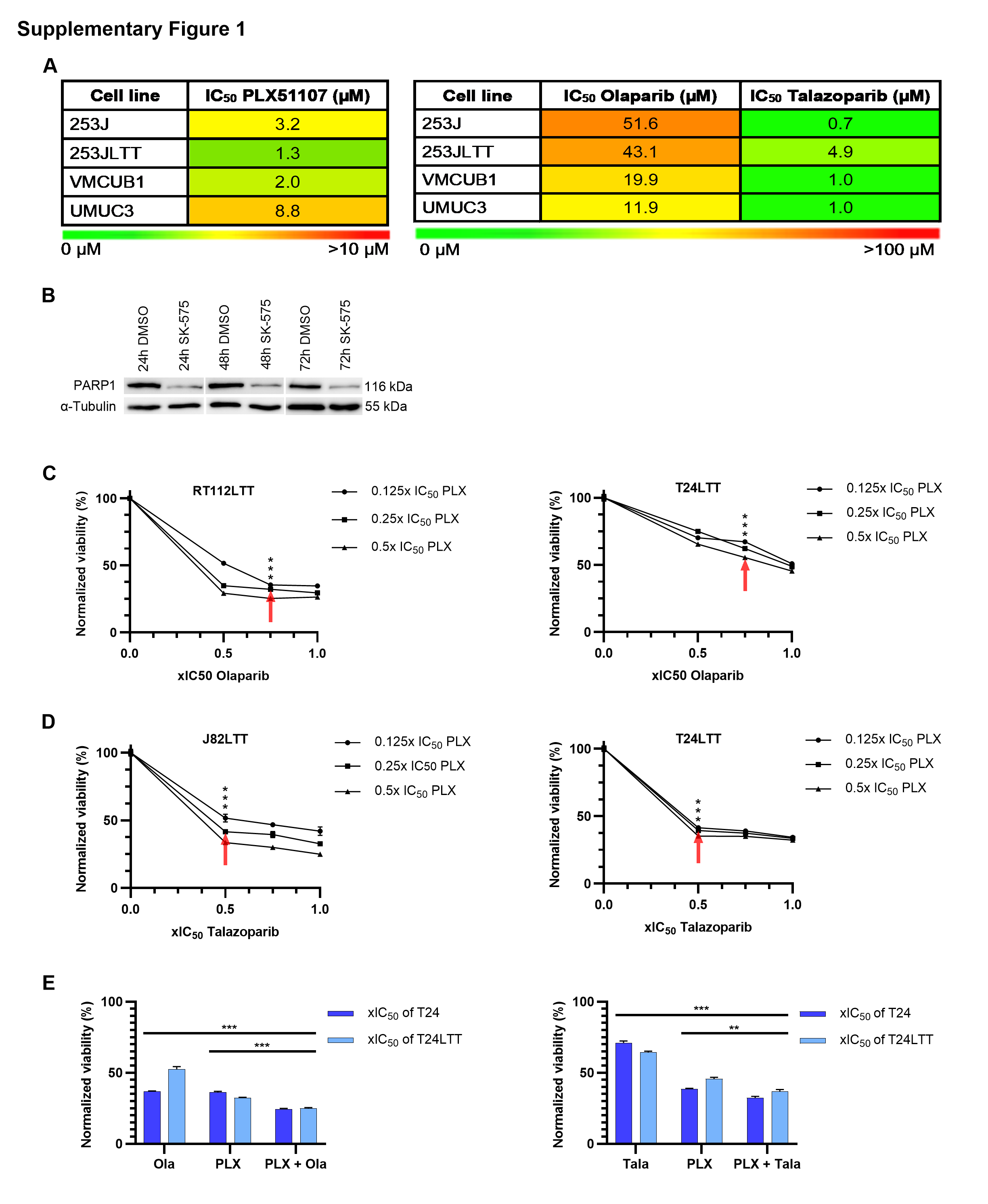

Supplement: Supplementary file 1 — Fig. S1. Additional IC50 values, PROTAC efficiency, selection of reduced dosages and normal toxicity of combination treatment. [file MOL2-20-779-s008.tif]

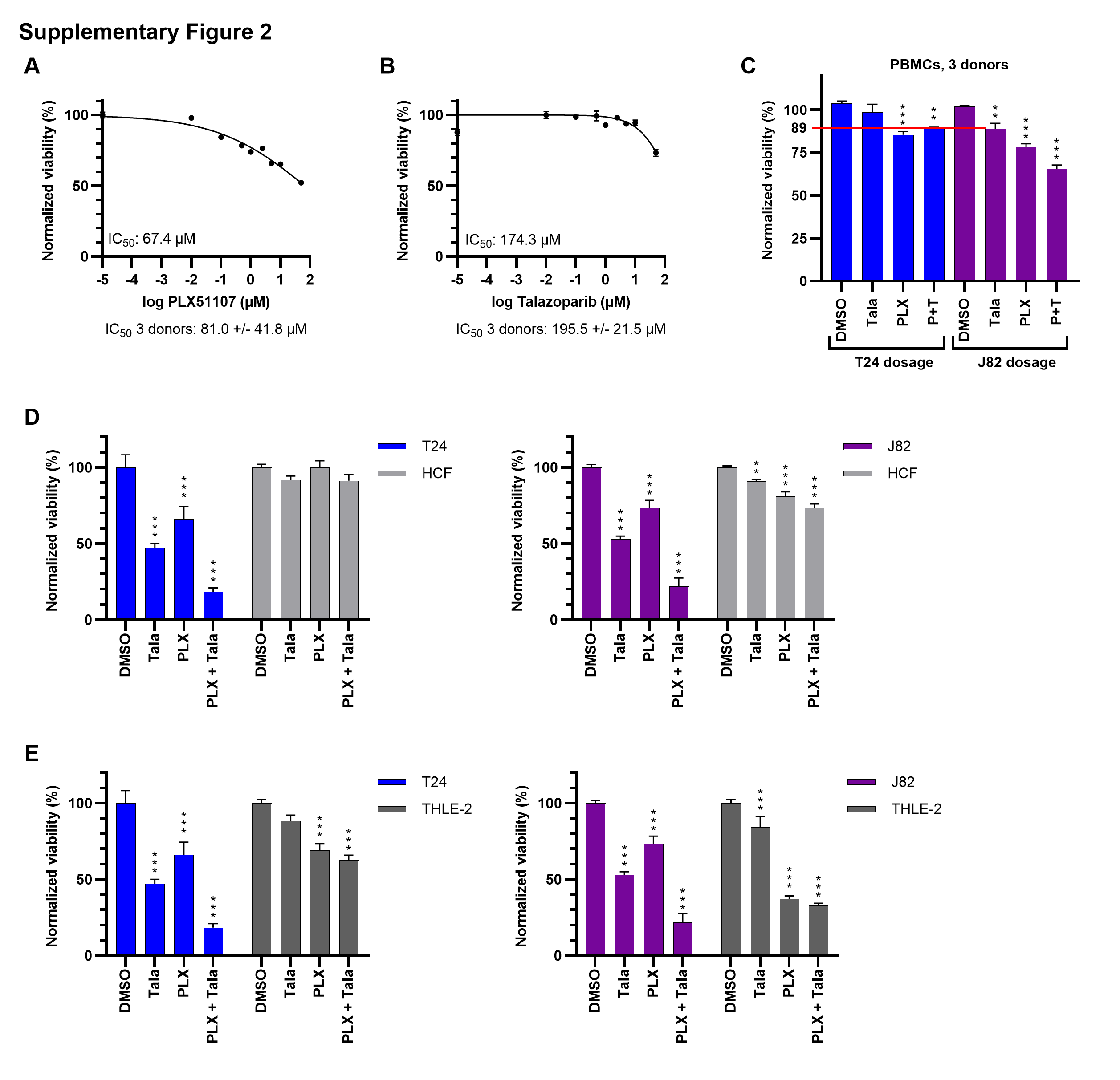

Supplement: Supplementary file 2 — Fig. S2. Additional analyses for normal toxicity. [file MOL2-20-779-s004.tif]

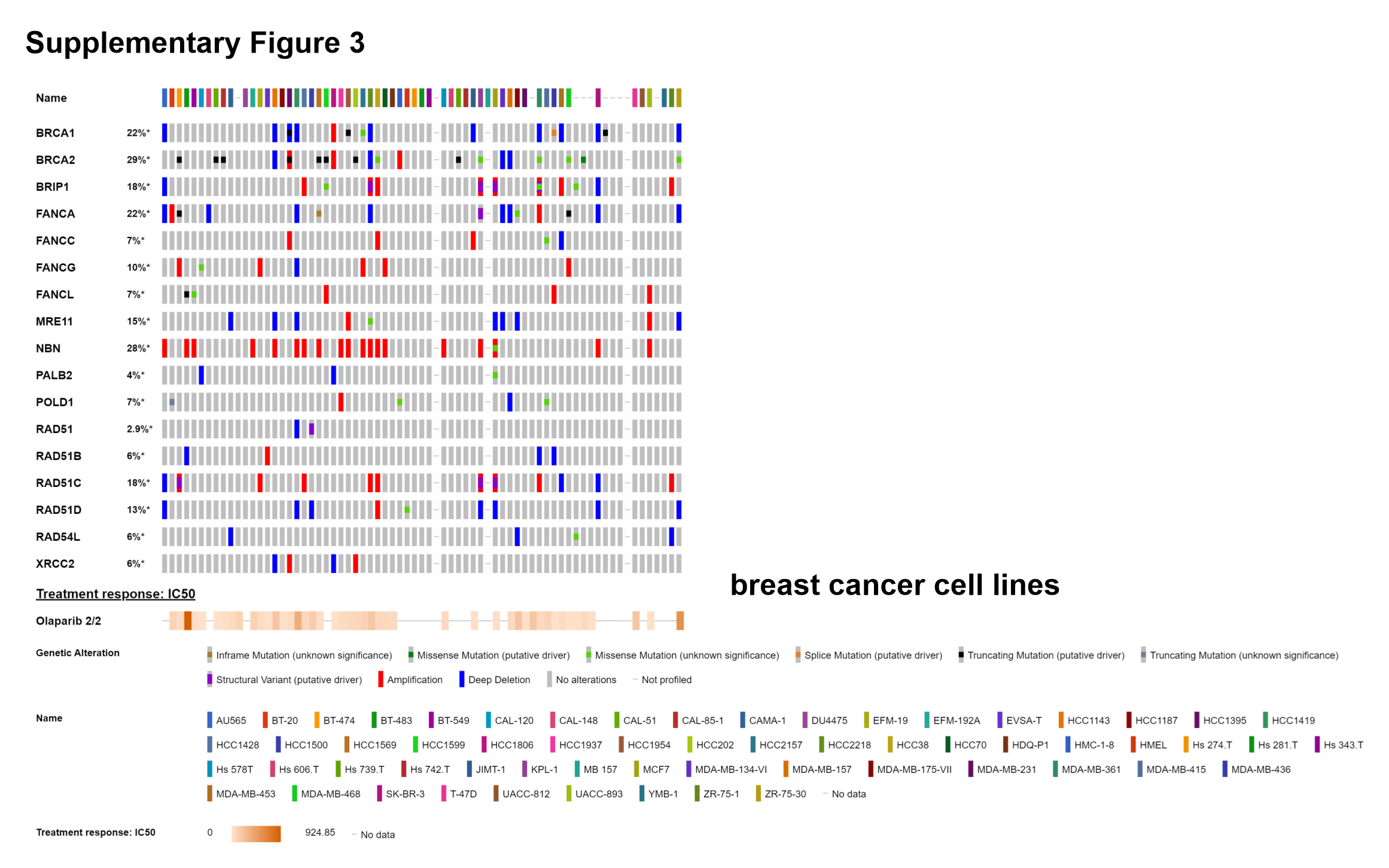

Supplement: Supplementary file 3 — Fig. S3. Genetic alterations in homologous recombination repair genes are frequent in breast cancer cell lines. [file MOL2-20-779-s003.tif]

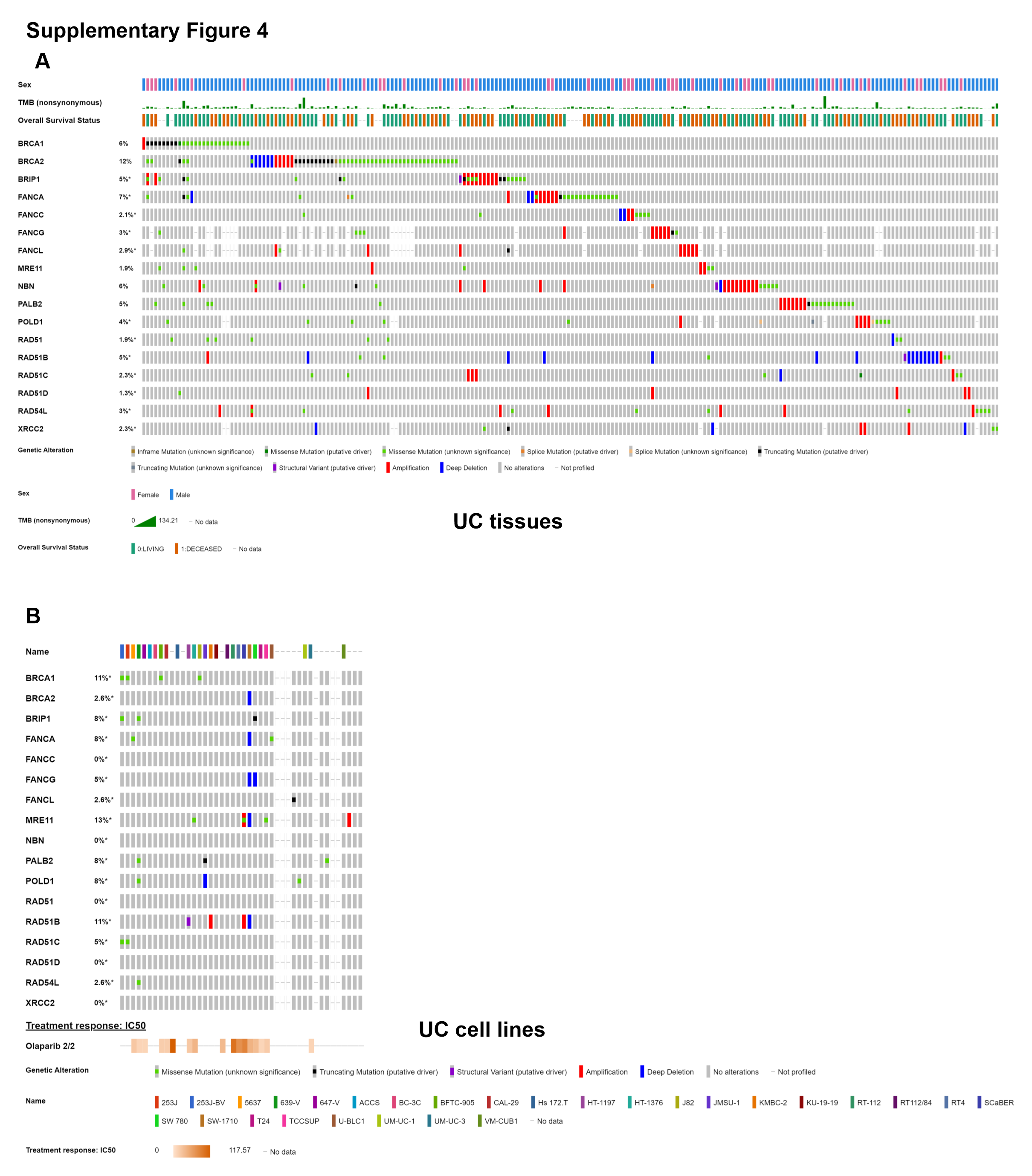

Supplement: Supplementary file 4 — Fig. S4. Genetic alterations in homologous recombination repair genes are less frequent in urothelial carcinoma. [file MOL2-20-779-s001.tif]

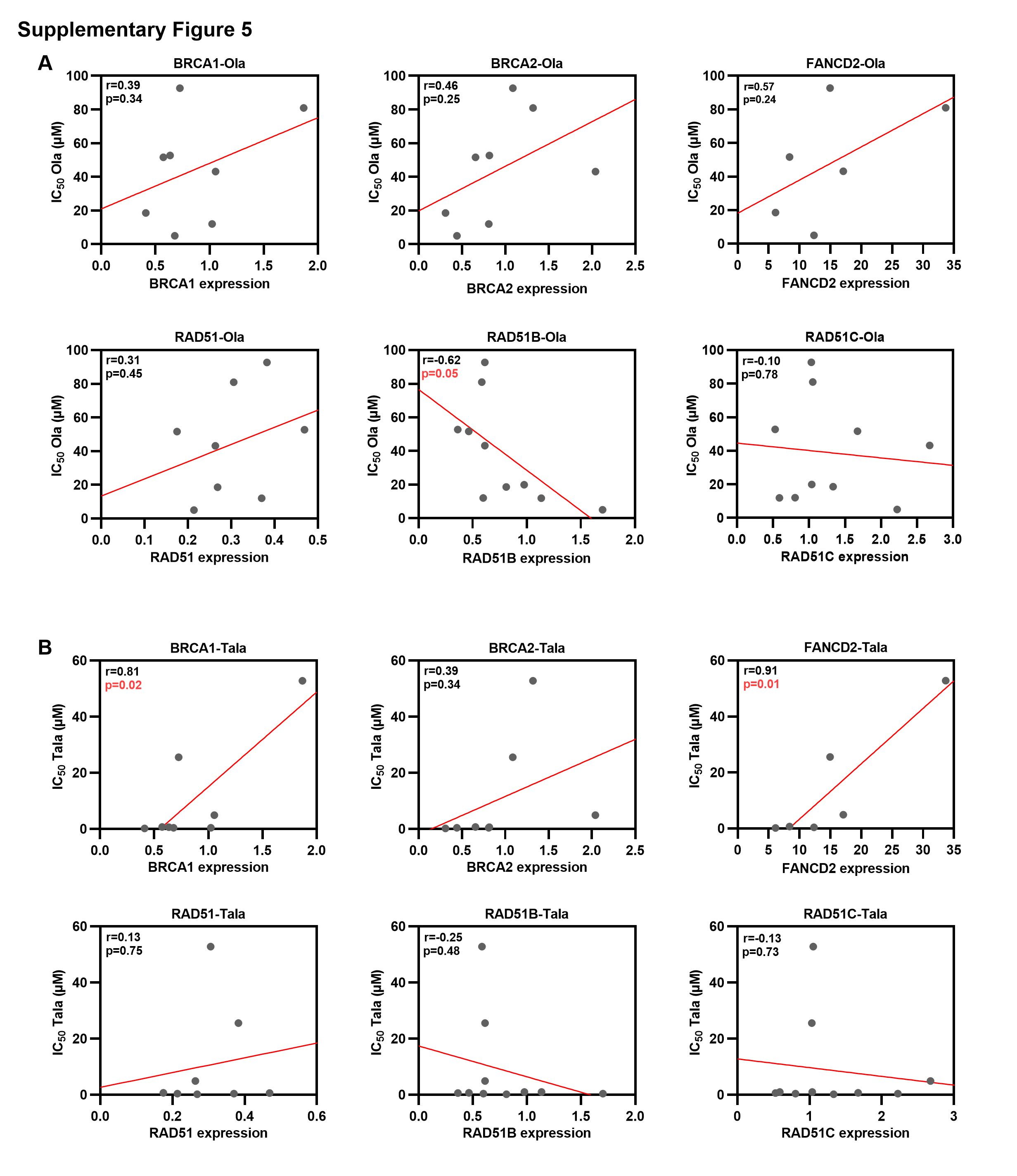

Supplement: Supplementary file 5 — Fig. S5. Correlation analysis for homologous recombination repair gene expression and PARP inhibitor response. [file MOL2-20-779-s011.tif]

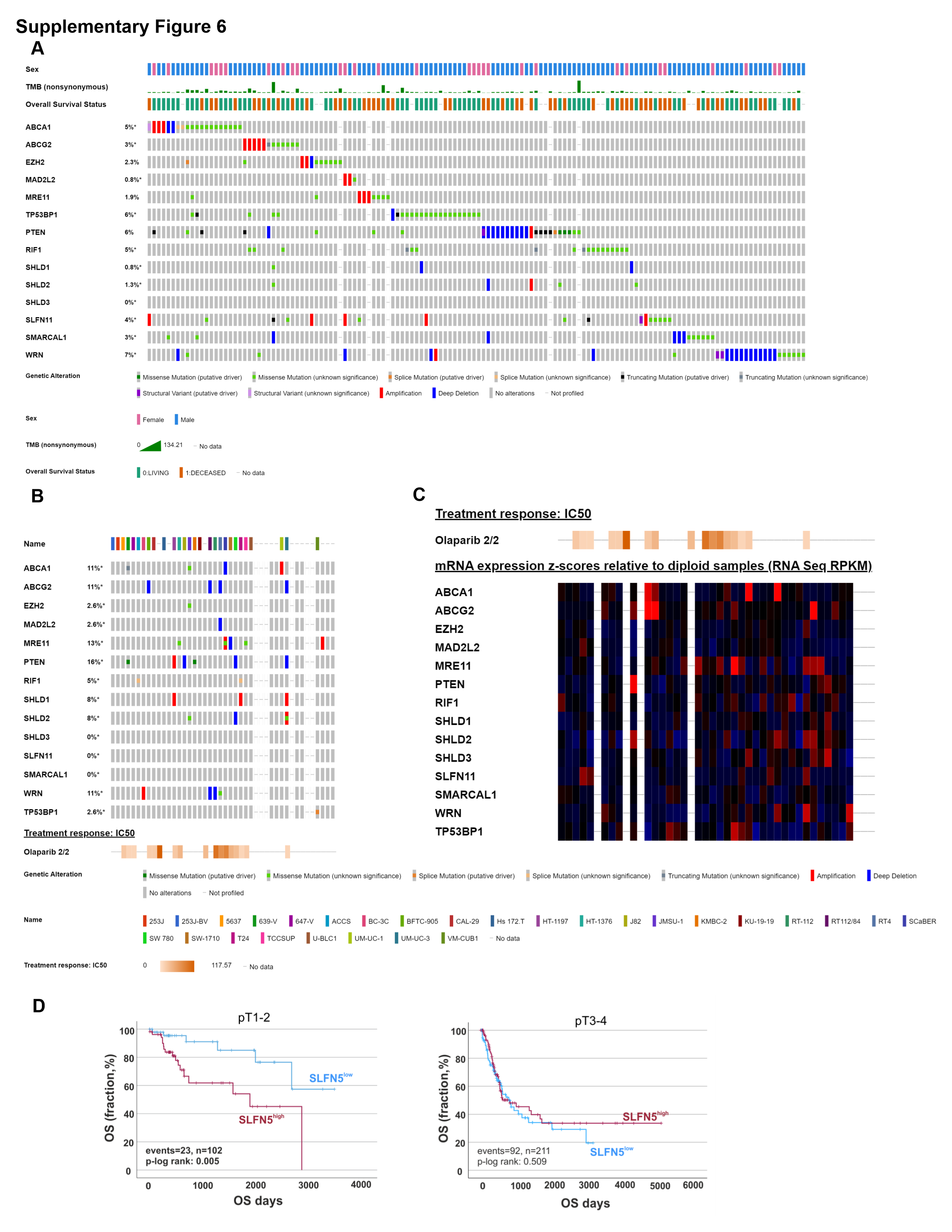

Supplement: Supplementary file 6 — Fig. S6. Alterations in putative PARP inhibitor resistance factors in urothelial carcinoma. [file MOL2-20-779-s010.tif]

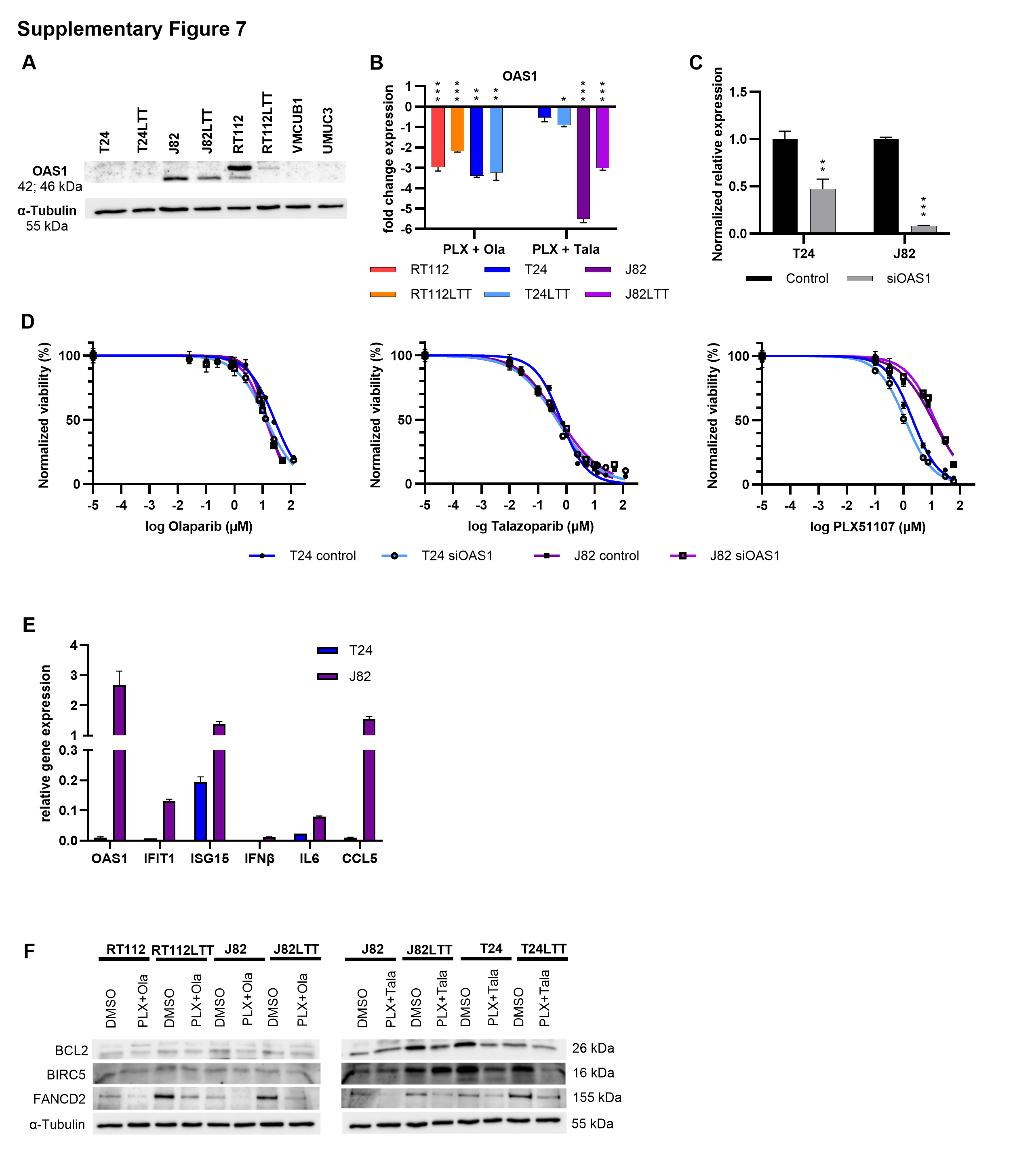

Supplement: Supplementary file 7 — Fig. S7. Further validation data for OAS1, cGAS‐STING and synergism factors. [file MOL2-20-779-s007.tif]
