# Supplementary material for: Improving PARP inhibitor efficacy in bladder cancer without genetic BRCAness by combination with PLX51107
Source: Mol Oncol. 2025 Nov 11;20(3):779–803. doi: 10.1002/1878-0261.70148 (PMC13042512; doi:10.1002/1878-0261.70148)

Raw data file: Raw western blot membranes.

Panel A

For Fig. 2 D: Cleaved PARP PLX+Ola

- 15 µg protein were loaded for each sample
- 7 µL of Marker #26616 (Thermo Scientific) were loaded

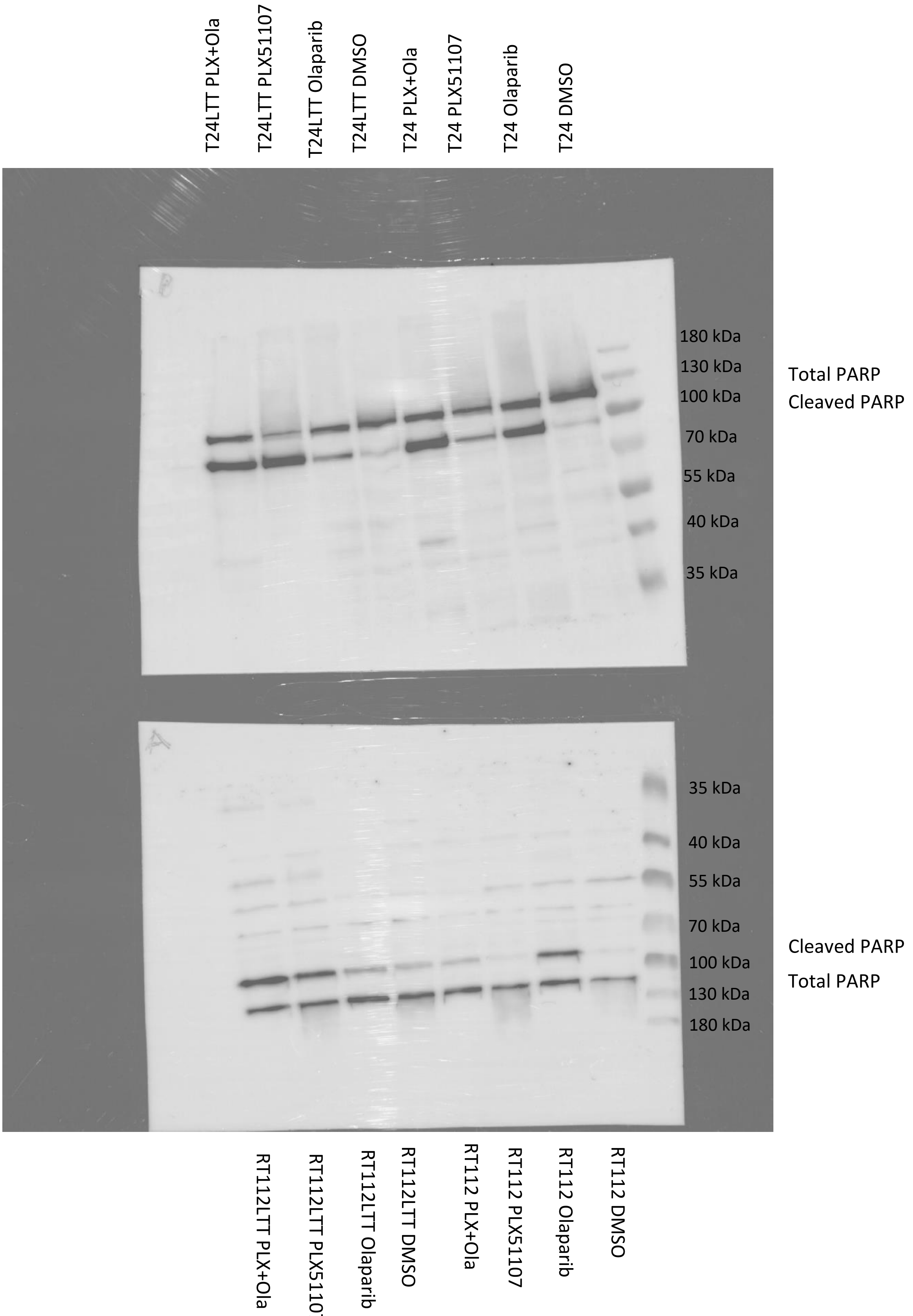

Panel B

For Fig. 2 D: Cleaved PARP PLX+Ola

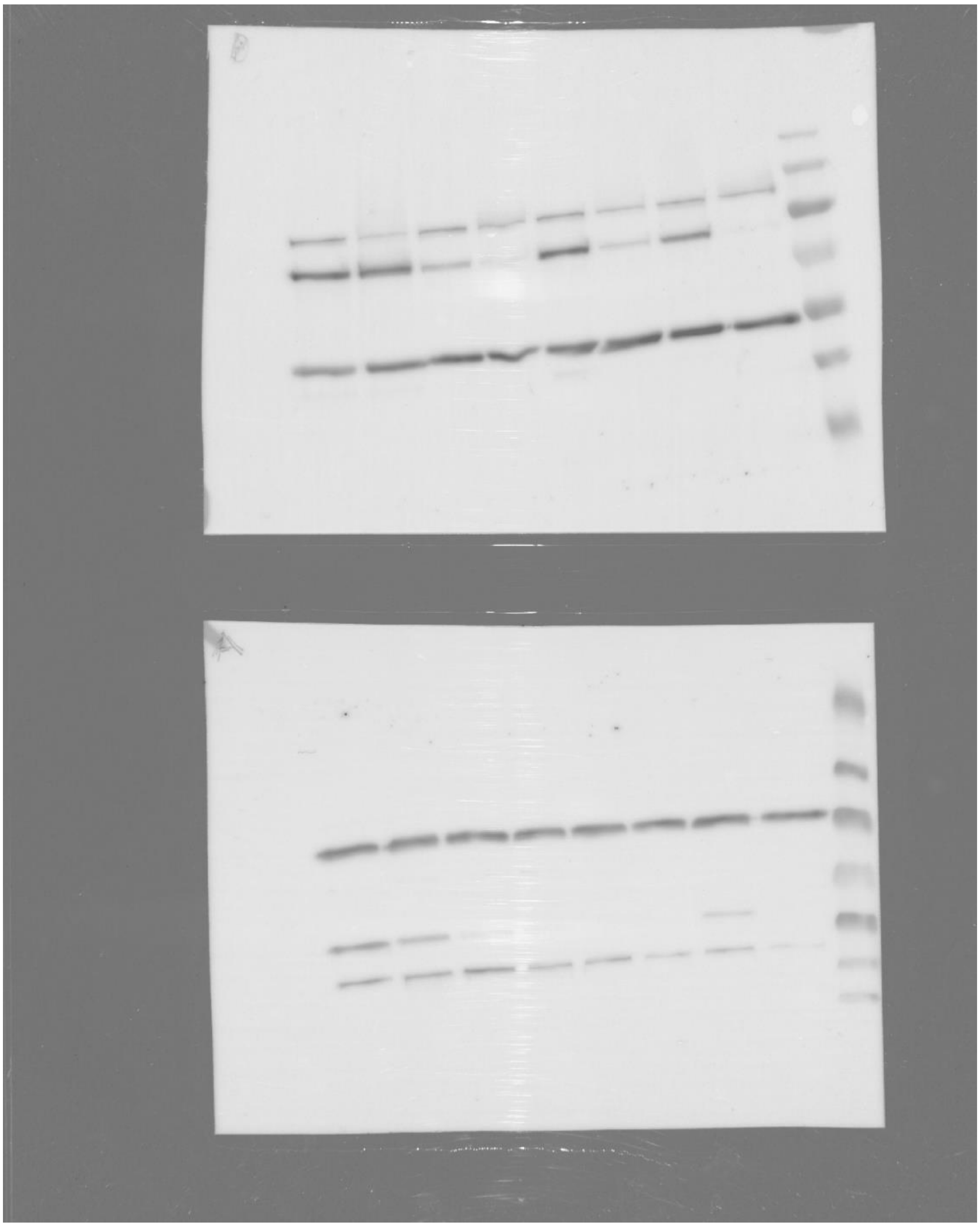

Panel C

For Fig. 2H: Cleaved PARP PLX+Tala

- 15 µg protein were loaded for each sample
- 7 µL of Marker #26616 (Thermo Scientific) were loaded

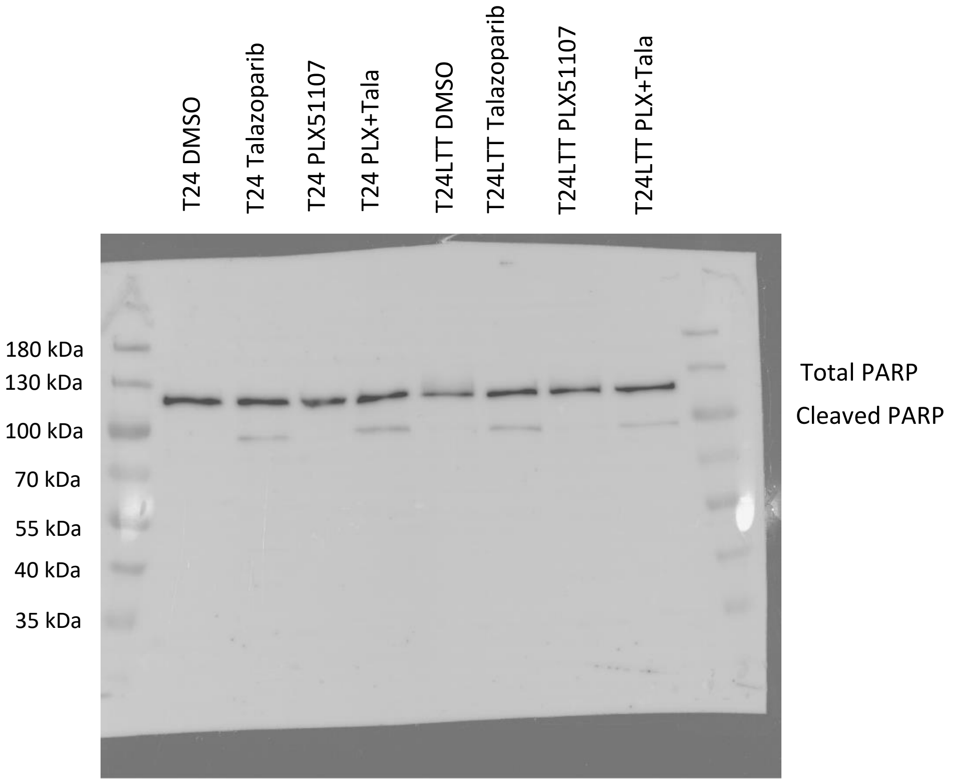

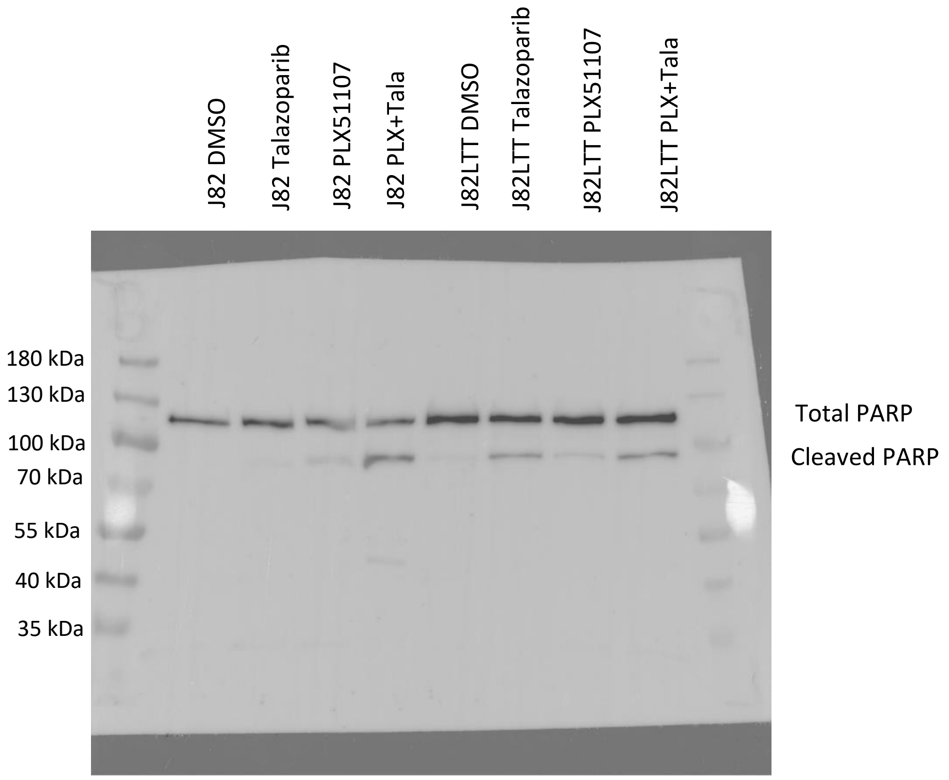

Panel D

For Fig. 2H: Cleaved PARP PLX+Tala

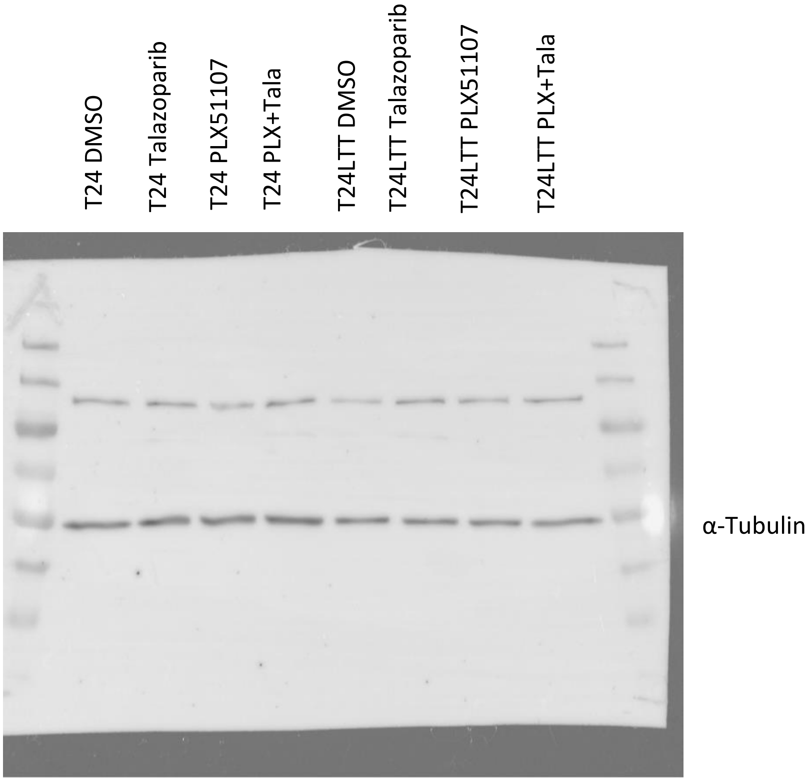

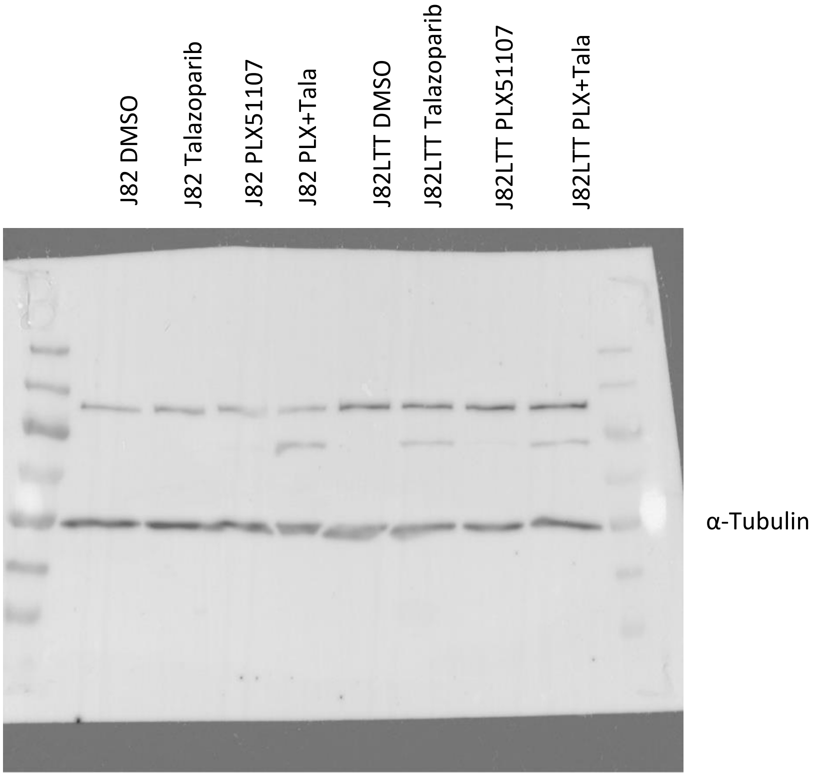

Panel E

For Fig. 3B: SLFN11 protein level across untreated UCCs

- 15 µg protein were loaded for each sample
- 7 µL of Marker #26616 (Thermo Scientific) were loaded
- The membrane was cut into pieces before antibody incubation and detection

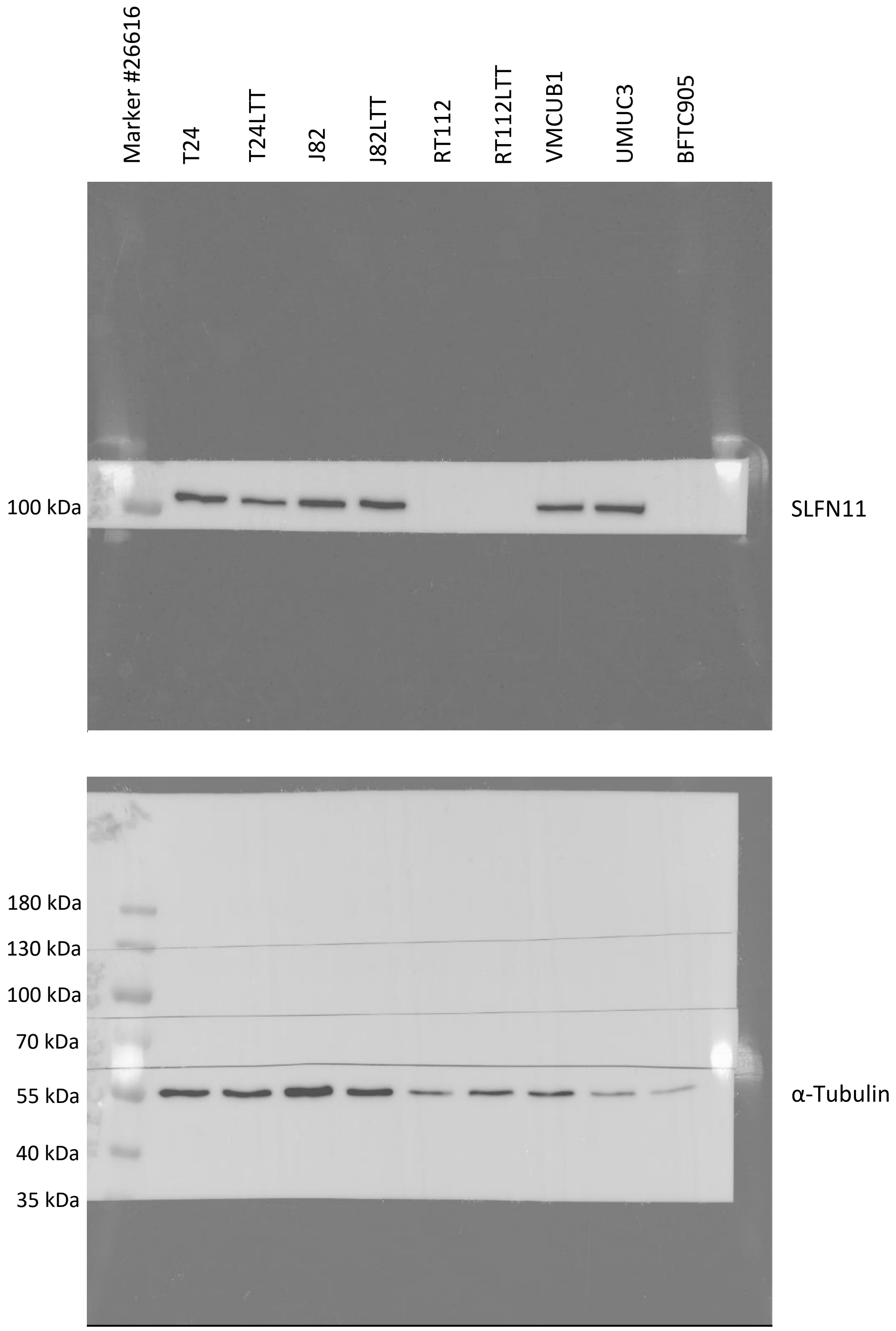

Panel F

For Suppl. Fig. 1B: T24 UCCs after treatment with PROTAC

- 15 µg protein were loaded for each sample
- 7 µL of Marker #26616 (Thermo Scientific) were loaded
- The membrane was cut into pieces before antibody incubation and detection

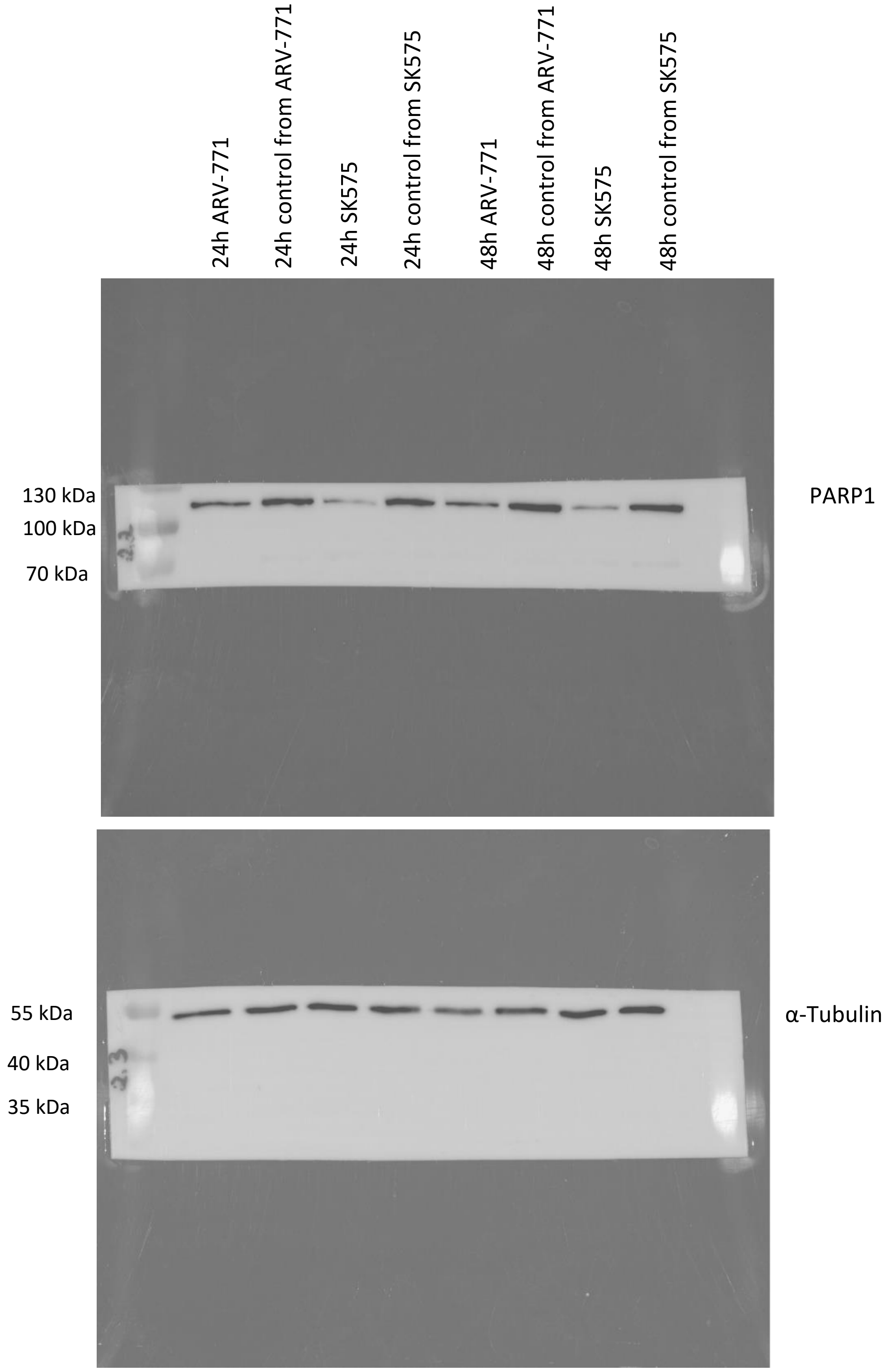

Panel G

For Suppl. Fig. 1B: T24 UCCs after treatment with PROTAC

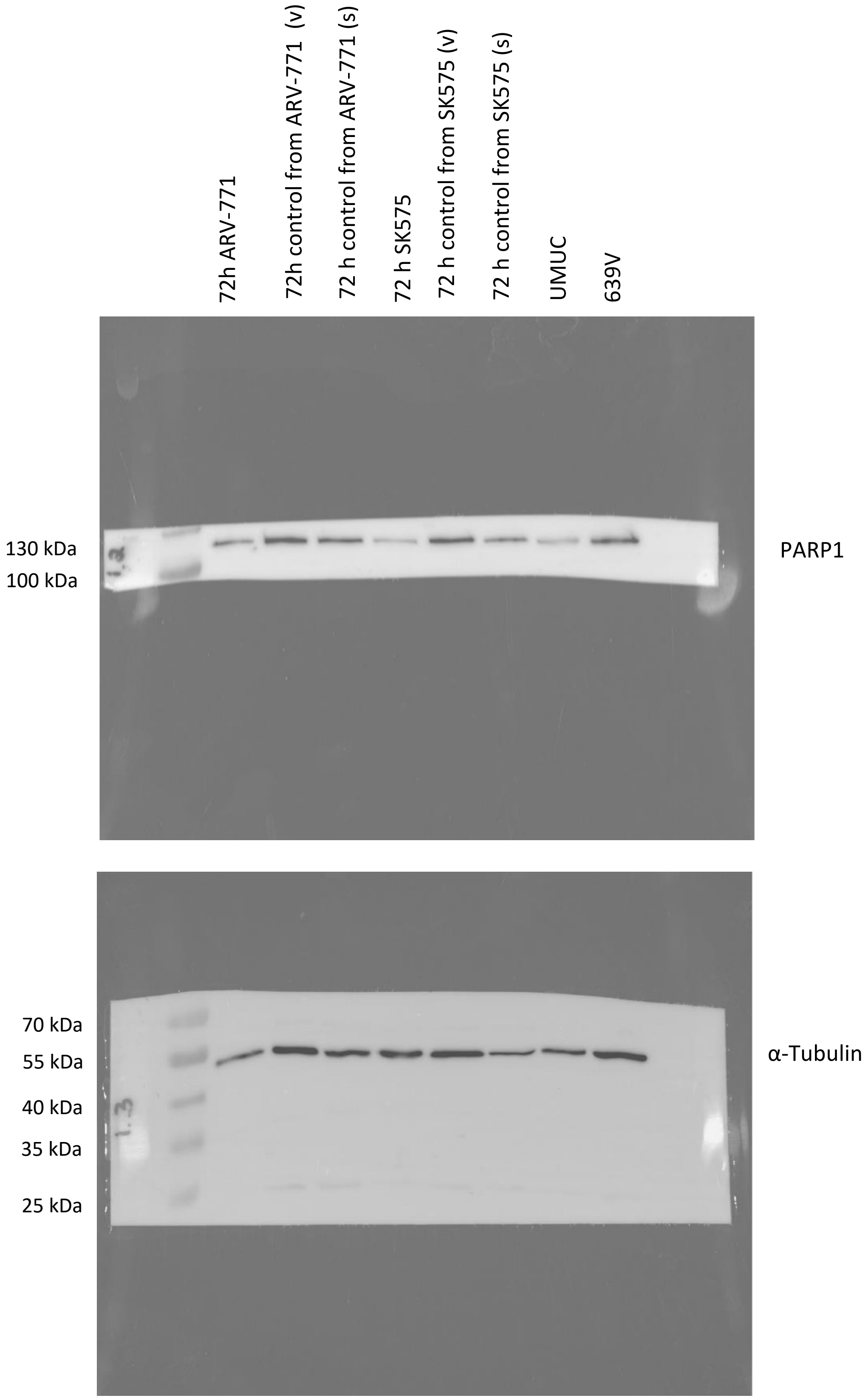

Panel H

For Suppl. Fig. 7A: OAS1 protein level across untreated UCCs

- 15 µg protein were loaded for each sample
- 7 µL of Marker #26616 (Thermo Scientific) were loaded
- The membrane was cut into pieces before antibody incubation and detection

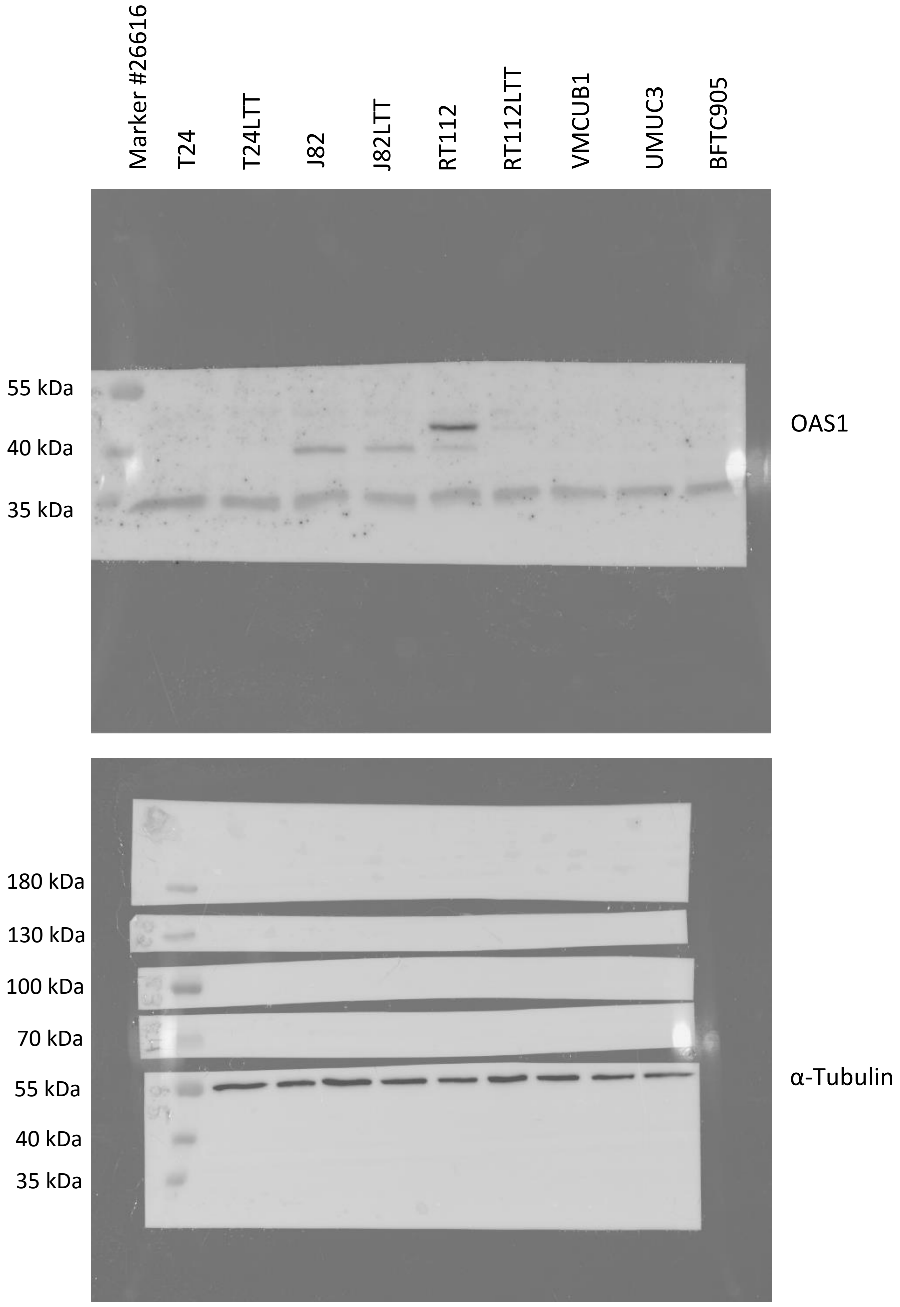

Panel I

For suppl. Fig. 7F: various protein levels across PLX+Ola and PLX+Tala treated UCCs

- 15 µg protein were loaded for each sample
- 7 µL of Marker #26616 (Thermo Scientific) were loaded
- The membrane was cut into pieces before antibody incubation and detection

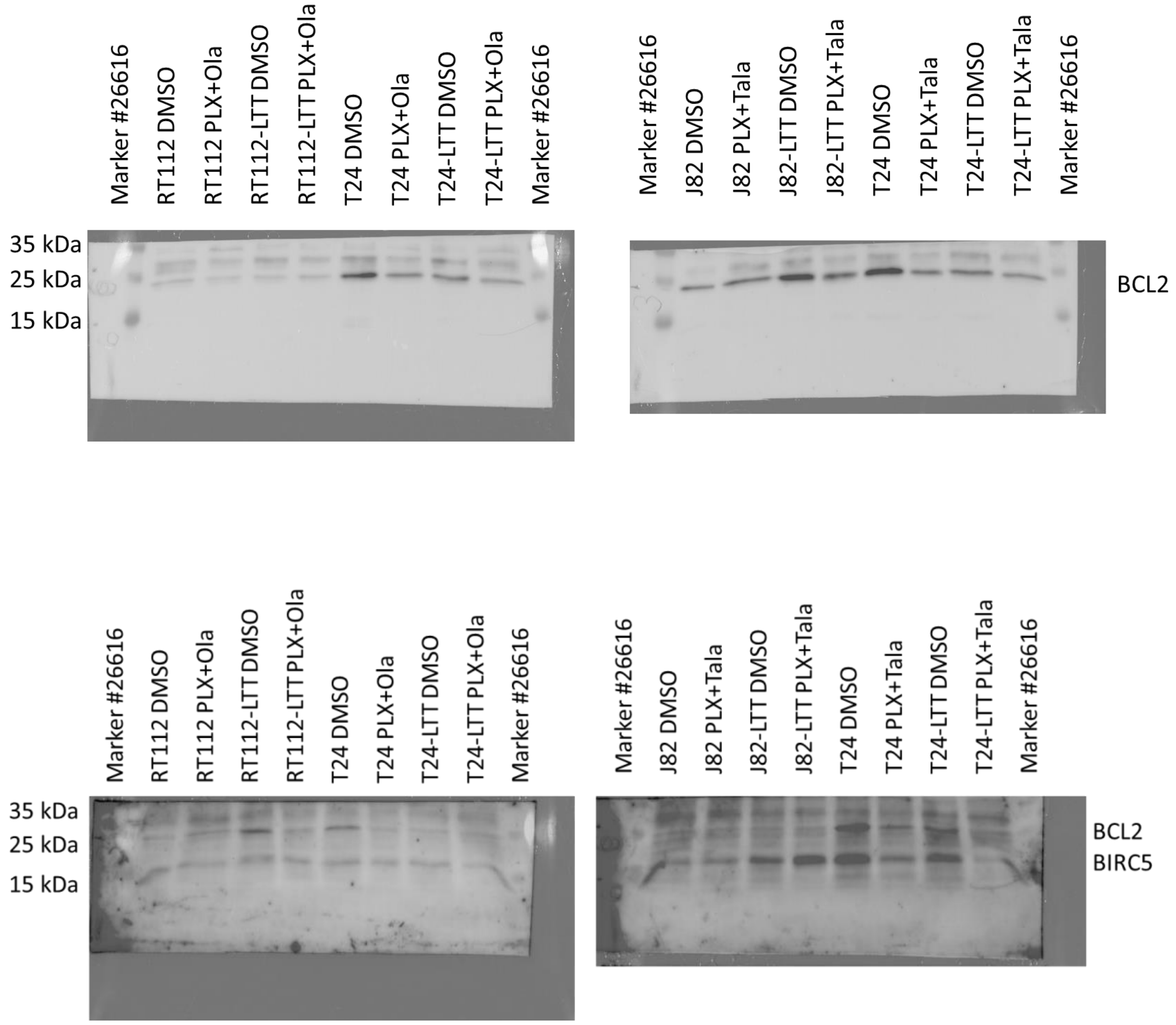

Panel I

For suppl. Fig. 7F: various protein levels across PLX+Ola and PLX+Tala treated UCCs

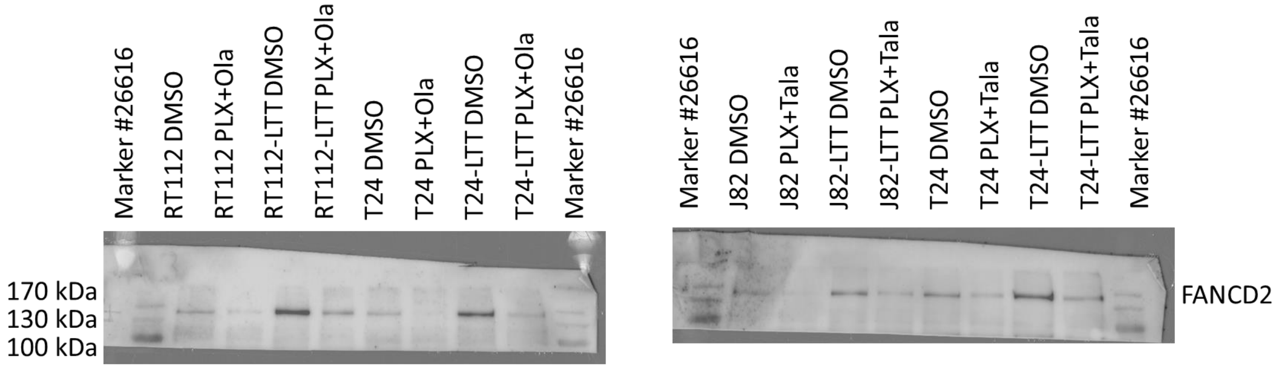

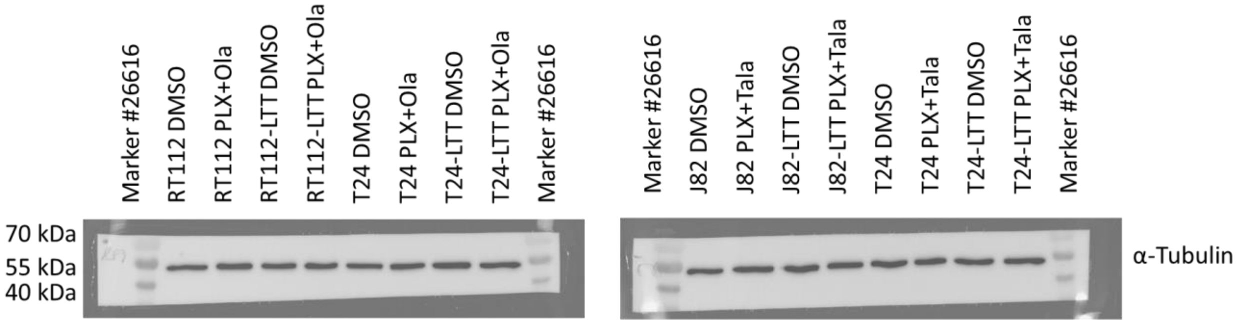

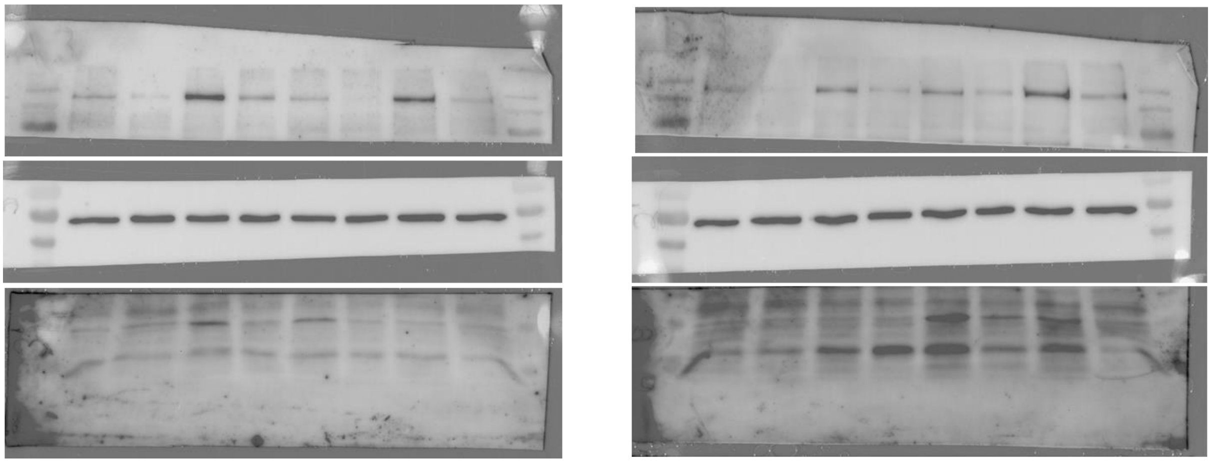

Supplement: Supplementary file 8 — Fig. S8. Raw western blot membranes. [file MOL2-20-779-s013.pdf]
